# Bridging scales: integrated multi-omics and deep phenotyping for climate resilience in crop plants

**DOI:** 10.3389/fpls.2026.1777294

**Published:** 2026-05-07

**Authors:** Sreekumar Anand, Sathanur Bhaskar Reddy, Niji Maheendran Shajini, Elizabeth Jose, Mahesh Santosh Shirsat, Ravindran Lalithambika Visakh, Uday Chand Jha, Rameswar Prasad Sah, Radha Beena

**Affiliations:** 1 Department of Genetics and Plant Breeding, Agronomic Research Station, Chalakudy, Kerala Agricultural University, Thrissur, Kerala, India; 2 Division of Genetics, Indian Agricultural Research Institute, New Delhi, India; 3 Department of Plant Breeding and Genetics, College of Agriculture, Vellayani, Kerala Agricultural University, Thiruvananthapuram, Kerala, India; 4 Department of Molecular Biology & Biotechnology, College of Agriculture, Vellayani, Kerala Agricultural University, Thiruvananthapuram, Kerala, India; 5 Department of Plant Breeding and Genetics, Indian Institute of Pulse Research, Kanpur, India; 6 Crop Improvement Division, ICAR-National Rice Research Institute, Cuttack, Odisha, India; 7 Department of Plant Physiology, College of Agriculture, Vellayani, Kerala Agricultural University, Thiruvananthapuram, Kerala, India

**Keywords:** climate resilience, cross validation, multiomics integration, multi-omics integration, prediction models

## Abstract

Global changes in agricultural and environmental systems will necessitate new crop research methodologies in the future years to ensure more effective use of natural resources and food security. The progress in next-generation sequencing has led to the emergence of multi-omics techniques as successful crop improvement strategies. Multi-omics studies using high-throughput techniques have been critical in understanding growth, senescence, yield, and biotic and abiotic stress responses in an array of crops. When multi-omics provide a high-resolution map of the molecular frameworks governing stress responses, advanced deep phenotyping systems can utilize advanced sensors to quantify dynamic physiological and morphological traits non-destructively. The systematic integration of these multi-layered datasets through association mapping and machine learning frameworks allows for the identification of superior alleles and regulatory hubs. Currently, the non-invasive imaging methods have effectively incorporated computer vision, machine learning, and deep learning components of AI. The use of machine learning and deep learning have progressively increased the effectiveness of data gathering and analysis. The supervised, unsupervised, and deep learning architectures have become effective tools for overcoming the genotype-to-phenotype gap, enabling more accurate predictions of yield and stress tolerance. Despite challenges related to data dimensionality, high infrastructure costs, and the need for standardized protocols, the convergence of these fields offers a robust architecture for predictive breeding. By linking microscopic molecular shifts to macroscopic field performance, integrated strategies accelerate the discovery of adaptive traits and the delivery of high-yielding, climate-smart cultivars. This review examines the revolutionary potential of combining deep phenotyping and multi-omics data for developing a thorough, high-throughput crop improvement strategy that can revolutionize crop breeding.

## 1 Introduction

Climate change is increasingly altering the environments in which crops are grown, with rising temperatures, erratic rainfall patterns, and more frequent episodes of drought and heat stress becoming common across agricultural regions worldwide. These changes threaten yield stability and food security, particularly in rainfed and semi-arid systems. As a result, breeding for climate resilience has become a central goal in crop improvement programs, requiring a deeper understanding of how plants respond to stress over time and across environments (Challinor et al., 2014; Wheeler and von Braun, 2013).

Traditional breeding approaches have relied largely on visual scoring and a limited number of manually measured traits, often recorded at one or two developmental stages. While such measurements remain valuable, they capture only static snapshots of plant performance and frequently fail to detect early or transient stress responses. In contrast, advances in genomics and related omics technologies have generated unprecedented volumes of molecular data, creating a growing mismatch between the resolution of genotypic information and the depth of phenotypic data available for selection (Yang et al., 2020; Cobb et al., 2013).

Multi-omics approaches including genomics, transcriptomics, proteomics, metabolomics, and ionomics have transformed crop research by enabling system-level analysis of stress responses. High-density genotyping and genome sequencing have accelerated gene discovery, allele mining, and trait dissection in major crops such as rice, wheat, and maize (Varshney et al., 2021; Kumar et al., 2024). Transcriptomic and metabolomic studies further demonstrate that drought, heat, and their combinations trigger extensive reprogramming of gene expression, hormone signaling, and primary and secondary metabolism (Zandalinas et al., 2021; Suzuki et al., 2014). However, molecular responses alone provide limited insight into agronomic performance unless they can be linked to measurable plant traits expressed under field conditions.

Deep phenotyping offers a way to bridge this gap by enabling non-destructive, high-frequency measurement of plant growth and physiology using imaging and sensor-based technologies. Platforms employing RGB, spectral, thermal infrared, fluorescence, and three-dimensional sensing can capture temporal dynamics of canopy development, pigment composition, water status, and stress responses with high precision (Araus and Kefauver, 2018; Fahlgren et al., 2015). The deployment of unmanned aerial vehicles and ground-based phenotyping systems has further extended these capabilities to large breeding trials, allowing thousands of plots to be monitored efficiently across environments (Volpato et al., 2021; Stutsel et al., 2021).

The true power of these technologies lies in their integration. Bridging the gap between a molecular candidate and the final yield of a field-grown crop requires sophisticated analytical strategies. Association mapping, specifically Genome-Wide Association Studies (GWAS), acts as a primary link, correlating specific genomic regions with the multi-dimensional phenotypes captured by deep phenotyping platforms. Machine Learning (ML) and Artificial Intelligence (AI) serve as the computational engine for this integration. These AI integrated ML models can handle the extreme dimensionality of multi-omics data, integrate with phenotyping platforms, filter out noise, and predict adaptive traits with high accuracy. Looking forward, the next generation of crop improvement will depend on interpretable AI that incorporates biological priors into its architecture. This review brings together recent progress in multi-omics technologies and deep phenotyping platform integration, with an emphasis on how it can connect molecular processes with physiological responses and field-level performance under climate stress. We examine emerging approaches that integrate high-dimensional omics data with temporally resolved phenotypic measurements to better capture stress-responsive traits, regulatory pathways, and adaptive responses relevant to crop improvement. In addition, by outlining current methodological developments alongside key integration strategies and unresolved challenges, this review seeks to offer both a conceptual framework and practical guidance for applying multi-omics–phenotyping approaches in the development of climate-resilient crops.

## 2 Multi-omics techniques

The advent of multi-omics technologies has revolutionized our comprehension of molecular mechanisms underlying critical traits across phylogenetically diverse plant species. Integrated multi-omics strategies encompassing genomics, transcriptomics, Proteomics, metabolomics, and ionomics have furnished unprecedented insights into the molecular frameworks governing crop resilience and productivity.

### 2.1 Genomics

The field of genomics, which involves comprehensive analysis of an organism’s complete genetic information, has become a pivotal force in revolutionizing crop improvement strategies. By leveraging advanced sequencing techniques and innovative methodological approaches, this scientific discipline is establishing a fundamentally new framework for enhancing crop performance under shifting climatic conditions (Kumar et al., 2024). The emergence of Next-Generation Sequencing (NGS) platforms has fundamentally transformed our capacity to decode plant genomic sequences with unprecedented speed and efficiency (Bolger et al., 2014). Moreover, with the advent of advanced long-read sequencing platforms, including those from Oxford Nanopore and PacBio, researchers can now obtain genome assemblies of enhanced precision and detect structural variants associated with stress tolerance at substantially lower error rates (Chen et al., 2019). Previously, non-coding DNA sequences were considered largely inconsequential. However, contemporary advances in genomic technology and theoretical frameworks have illuminated these regions as invaluable reservoirs for novel breeding objectives (Pourkheirandish et al., 2020). These Genomic insights have revolutionized precision genome editing, especially through CRISPR-Cas9, which facilitates the deletion, insertion or replacement of genes governing plant stress responses (Li and Xia, 2019). Notably, CRISPR-mediated modification of pivotal transcription factor genes (DREB and NAC families) has demonstrably augmented plant resilience against abiotic stressors such as drought, salinity, and thermal extremes (Kim et al., 2018; Rai et al., 2023).

Genome-wide association studies (GWAS) and quantitative trait locus (QTL) mapping, leveraging high-density single-nucleotide polymorphism (SNP) and structural variant datasets, facilitate the precise identification of genomic regions correlated with stress tolerance phenotypes, thereby expediting marker-assisted selection strategies in crop improvement programs (Tyagi et al., 2024). GWAS enables researchers to systematically map SNPs to key agronomic stress tolerance traits, including drought endurance, salinity resistance, and heat tolerance (Chen et al., 2023; Li et al., 2023). The SNP markers offer several compelling advantages, including their high abundance throughout the genome, biallelic nature, remarkable reproducibility, low mutation rates, and compatibility with high-throughput automated platforms (Verma et al., 2015). Of particular significance are SNPs residing within protein-coding regions, which may directly influence agronomic traits by inducing alterations in the amino acid composition of the encoded protein; such variants are consequently designated as functional SNPs or perfect markers. Owing to these distinctive attributes, SNP-based genotyping has witnessed widespread and accelerating adoption across a broad spectrum of economically important crop species with particular emphasis on the elucidation of stress tolerance mechanisms (Jain et al., 2013; Yadava et al., 2023). Moreover, rapid progress in next-generation sequencing technologies has substantially reduced the costs associated with DNA sequencing, rendering genotyping-by-sequencing (GBS) a practically viable and efficient approach for species characterized by high genetic diversity and large genome size. GBS enables large-scale sample multiplexing through barcoding, facilitating high-throughput SNP genotyping at a significantly reduced cost per sample compared to high-density SNP chip arrays (Elshire et al., 2011; Singh et al., 2020). The limitation of non-uniform genome coverage inherent to GBS has been effectively addressed by restriction site-associated DNA (RAD) sequencing, a methodology encompassing sequential steps of restriction enzyme digestion for reduced genome representation, adapter ligation, PCR amplification, and subsequent sequencing. For instance, RAD-GBS has enabled the identification of 6920 genes under heat and drought stresses in pearl millet (Sun et al., 2020; Srivastava et al., 2022).

An additional cost-effective approach for routine genotyping and genetic analysis is skim sequencing, which utilizes low-volume Illumina Nextera chemistry and accommodates the multiplexing of up to 960 samples within a single library through dual-index barcoding. This dual-index barcoding strategy offers considerable flexibility, allowing the number of multiplexed samples to be adjusted according to data requirements, with the potential to scale beyond 3,072 samples. Sequencing coverage ranging from 1× to as low as 0.01× per sample enables the detection of chromosome dosage variation, identification of aneuploidy, and karyotypic characterization of introgression lines from skim-sequencing data (Adhikari et al., 2022).

Extensive genomic research conducted across multiple biological levels has advanced our understanding of the molecular mechanisms underlying plant adaptation to stress. Numerous quantitative trait loci (QTLs) have been identified in association with diverse traits under varying stress conditions. Notably, key QTLs such as *Saltol* on chromosome 1, along with functionally characterized genes including *SKC1* and *OsHKT1-5*, have been implicated in the regulation of ion transport and exclusion, effectively preventing Na^+^ toxicity in shoot tissues and thereby conferring salt tolerance in rice (Javaid et al., 2025). Candidate genes within identified QTL regions are typically characterized on the basis of functional annotation or sequence homology, and frequently encompass transcription factors including DREB, NAC, MYB, and bZIP families, which serve as master regulators orchestrating stress-responsive gene networks (Ullah et al., 2026). Pangenome analyses have further enabled comparative evaluation of diverse haplotypes, facilitating the identification of superior alleles associated with beneficial traits such as osmotic adjustment (Khan et al., 2020). Furthermore, regulatory variants are increasingly being targeted in breeding strategies to mitigate the growth penalties commonly associated with constitutive stress-responsive gene expression.

Functional validation of identified associations is a critical step, achievable through targeted gene knockout approaches employing CRISPR-Cas9 technology or through overexpression studies aimed at enhancing gene activity. For instance, overexpression of the *Arabidopsis NHX1* gene has been demonstrated to confer improved tolerance to both salinity and oxidative stress in mungbean (Kumar et al., 2017). Complementary to this, expression analysis serves as an effective validation strategy by elucidating spatiotemporal gene expression patterns under stress conditions. Furthermore, the development of near-isogenic lines (NILs) and independent mapping populations provides robust genetic frameworks for the rigorous validation of stress-associated loci and their functional significance.

The integration of pan-genomic approaches with structural and copy number variations has elucidated pivotal adaptive mechanisms conferring stress tolerance across diverse germplasm assemblages (Liu et al., 2020). Moreover, comparative genomic approaches have elucidated the evolutionary underpinnings of stress resilience mechanisms. For instance, a comprehensive genomic comparison between halophytes and glycophytes has illuminated distinctive genomic attributes, particularly gene families associated with salinity tolerance, which play pivotal roles in their ecological adaptation strategies (Dassanayake et al., 2011; Kajrolkar, 2025).

**Figure 1:**
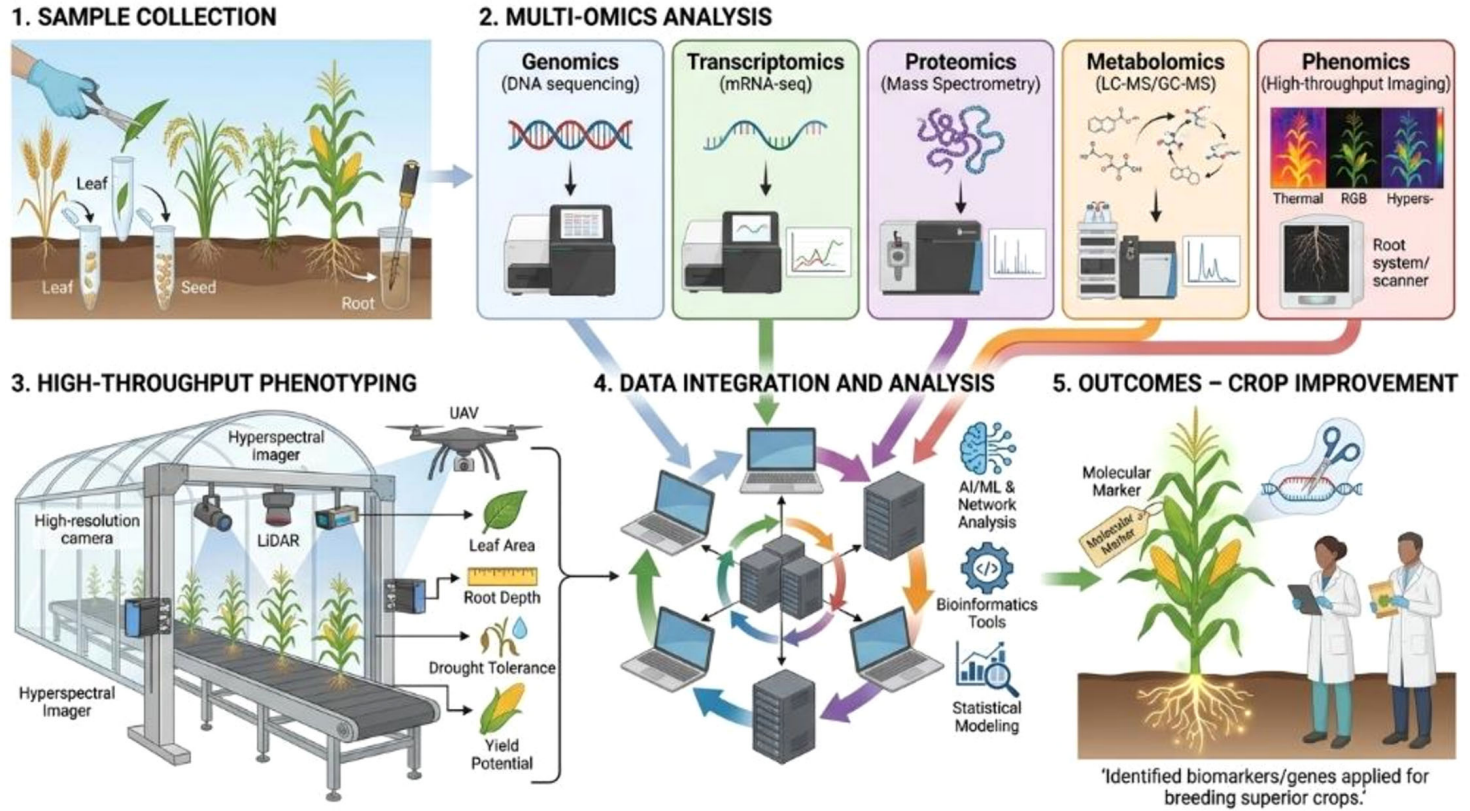
Integration of multi-omics techniques with high-throughput phenotyping for accelerating crop improvement. The workflow illustrates a five-stage pipeline: (1) Sample Collection (2) Multi-Omics Analysis- samples are subjected to genomics (DNA sequencing), transcriptomics (mRNA sequencing; mRNA-seq), proteomics (mass spectrometry), metabolomics (liquid chromatography-mass spectrometry/gas chromatography-mass spectrometry; LC-MS/GC-MS), and phenomics (high-throughput imaging including thermal, RGB, and hyperspectral modalities). (3) High-Throughput Phenotyping-plant phenotypic traits, including leaf area, root depth, drought tolerance, and yield potential, are captured using ground-based platforms equipped with hyperspectral imagers, high-resolution cameras, and LiDAR (light detection and ranging) sensors, as well as unmanned aerial vehicles (UAVs). (4) Data Integration and Analysis - multi-omics and phenomics datasets are integrated and analysed using artificial intelligence/machine learning (AI/ML), network analysis, bioinformatics tools, and statistical modeling. (5) Outcomes Crop Improvement identified biomarkers and candidate genes are deployed through molecular marker-assisted selection and precision breeding to develop superior, stress-resilient crop varieties.

Additionally, epigenomics data integration represents another critical dimension, elucidating molecular phenomena arising from DNA methylation and histone modifications that modulate gene expression independent of DNA sequence alterations (Xie et al., 2022). Recent technological breakthroughs, notably the CUT & Tag (Cleavage Under Targets and Tagmentation) technique, have enabled high-resolution mapping of these epigenetic modifications (Fu et al., 2024). Comprehensive epigenetic investigations utilizing advanced methodologies will undoubtedly address numerous unresolved questions, with the collective insights significantly contributing to enhanced stress resilience strategies. A holistic integration of genomics with other omics modalities, alongside cutting-edge high-throughput phenotyping methodologies, furnishes comprehensive insights for crop enhancement programs. The systematic integration and analysis of these multi-layered datasets culminate in the generation of superior climate-resilient crop varieties (**Figure 1**).

### 2.2 Transcriptomics

Transcriptomics entails the comprehensive analysis of cellular or organismal RNA transcripts, elucidating dynamic gene expression patterns with unprecedented resolution (Younas et al., 2025). Two predominant approaches currently define the transcriptomic landscape. Microarray analysis constitutes the first methodology, leveraging sequence-specific hybridization to assess RNA expression levels from known genetic sequences (Chaddad et al., 2023). Conversely, RNA sequencing (RNA-Seq) utilizes high-throughput sequencing technologies to systematically profile the complete transcriptional repertoire within biological samples, exhibiting exceptional applicability and facilitating transcriptome assembly in non-model species lacking established reference genomes (Wang and Huo, 2022). Coordinated multi-gene expression critically determines plant stress tolerance, and plants activate distinct gene expression profiles in response to diverse environmental stressors, thereby establishing integrated signaling architectures that confer stress resilience (Javaid et al., 2022). RNA-Seq surpasses microarrays in both sensitivity and coverage, revealing novel transcripts and rare RNA molecules essential for stress tolerance mechanisms. Transcriptomic profiling through RNA-seq has facilitated the identification of key transcription factors that orchestrate stress resilience pathways in major crop species such as Arabidopsis (Seok et al., 2020), soybean (Chen et al., 2016), rice (Kumar et al., 2022), and wheat (Guo et al., 2020).

Emerging single-cell RNA sequencing (scRNA-seq) platforms have enabled the deciphering of cell-type-specific transcriptional dynamics, revealing remarkable heterogeneity in stress adaptation mechanisms at the cellular level (Singh et al., 2024). Notably, single-cell transcriptomic profiling of Arabidopsis root tissues exposed to salinity stress revealed cell-type-resolved gene expression patterns, each corresponding to discrete adaptive mechanisms in stress mitigation (Ryu et al., 2019). Non-coding regulatory RNAs, including microRNAs and long non-coding RNAs, fine-tune gene expression through post-transcriptional and epigenetic regulation. Concurrently, pioneering scRNA sequencing technologies are elucidating cell-type-resolved transcriptional dynamics, thereby revealing remarkable cellular heterogeneity underlying stress adaptation strategies (Jain, 2024). Comprehensive Genome-wide transcriptomic profiling has unveiled stress-responsive long non-coding RNAs and small RNA species, including microRNAs and small interfering RNAs, which serve as pivotal regulators of gene expression and stress-induced signaling cascades (He et al., 2019). Thus, transcriptomics helps in preparing gene regulatory networks (GRNs), offering an integrated framework for understanding how plants synchronize complex molecular pathways under stress conditions (Meraj et al., 2020). The advent of spatial transcriptomics has revolutionized plant research, enabling scientists to map stress-responsive gene expression across specific root zones and leaf tissues with remarkable precision. These studies unveil how plants integrate localized cellular responses with organism-wide stress signaling networks (Yin et al., 2023).

Transcriptome-wide association study (TWAS) methodologies are designed to delineate the genetic determinants of phenotypic variation through the mediation of gene transcription. In this framework, predictive models of gene expression are computationally trained as functions of genetic variants and subsequently integrated with GWAS data. This post-GWAS analytical approach identifies gene-trait associations with enhanced interpretability, thereby facilitating downstream functional genomics investigations and the establishment of genetically anchored resources for comprehensive trait dissection (Evans et al., 2024). Substantial research efforts have been directed toward elucidating stress resilience mechanisms in crop species. Through TWAS, a gene encoding the Increased Salt Tolerance1-Like1 (ISTL1) protein, putatively involved in intracellular protein and membrane trafficking, was identified as the most compelling candidate causal gene in maize. A panel of maize nested near-isogenic lines harboring the *ISTL1* genomic region derived from eight donor parents was subsequently evaluated for leaf cuticular stomatal conductance, corroborating the association between leaf cuticular conductance and *ISTL1* through haplotype-based association analysis. Collectively, these findings provide valuable insights into the role of regulatory variation in maize leaf cuticle development and are anticipated to assist plant breeders in the development of drought-tolerant maize varieties tailored to target environments (Lin et al., 2022).

A recent investigation in rice has identified *OsDREB1C*-H3 as a superior haplotype conferring enhanced drought tolerance, with its presence consistently observed across all examined drought-tolerant rice varieties while remaining absent in susceptible genotypes. Additional drought-responsive genes validated in rice include *OsGSK1*, *OsDSR2*, and *ZFP182*, further expanding the genetic framework underlying drought adaptation. Building upon these findings, researchers are actively developing haplotype-specific near-isogenic lines (Haplo-NILs) through the introgression of superior haplotype combinations into elite genetic backgrounds. Notably, donor genotypes harboring complementary haplotype combinations, such as *OsDREB1C*-H3 and *DSM3*-H4, are currently being utilized in breeding programs aimed at developing high-yielding, drought-tolerant rice varieties suited to water-limited environments (Singh et al., 2024).

Candidate gene prioritization can be accomplished through a range of complementary strategies, including statistical colocalization, mediation analysis, gene regulatory network analysis, and integrative machine learning approaches such as expression quantitative trait loci (eQTL) integration. In cotton, the colocalization of eQTLs has proven instrumental in refining extensive pools of candidate genes within QTL regions to a limited number of functionally relevant candidates associated with salinity tolerance (Han et al., 2022). Furthermore, the integration of joint TWAS with locus-level colocalization analysis has demonstrated enhanced specificity and sensitivity in implicating biologically meaningful genes underlying complex traits (Hukku et al., 2022).

### 2.3 Proteomics

Climate-driven abiotic stresses such as drought, salinity, and heat induce substantial alterations in protein abundance, localization, and post-translational modifications in plants. Proteomics provides a robust analytical platform for analyzing these global protein changes, revealing functional stress responses that transcriptomic approaches alone cannot fully predict (Kosova et al., 2011). Cutting-edge proteomic methodologies, particularly mass spectrometry (MS) based platforms, have enabled the comprehensive identification, quantification, and functional characterization of proteins mediating plant stress responses. Furthermore, integrated thermal proteome profiling has emerged as a valuable tool for elucidating how temperature fluctuations influence protein stability and molecular interactions during stress conditions (Jensen et al., 2023). Additionally, protein-protein interaction (PPI) network methodologies, including yeast two-hybrid screening, bimolecular fluorescence complementation, and co-immunoprecipitation coupled with MS, have been instrumental in deciphering stress signaling cascades and adaptive responses in plants (Barah et al., 2016). These proteomic analyses have proven invaluable for identifying stress-induced post-translational modifications such as phosphorylation, ubiquitination, and sumoylation that critically regulate protein stability, activity, and interactions during stress adaptation. As an example, MAPK phosphorylation serves as a critical molecular switch, transducing environmental signals into specific cellular responses (Singh et al., 2022; Sharma et al., 2023).

Considerable research efforts have been directed toward crop improvement for enhanced stress tolerance through proteomics-based approaches. A nano-LC-MS/MS-based leaf proteome analysis investigating sorghum responses to drought stress identified a total of 3,927 proteins, with 46, 36, 35, and 102 Differentially Abundant Proteins (DAPs) detected in the S4, S4-1, T14, and T33 varieties, respectively. Drought-tolerant genotypes exhibited upregulation of the TCA cycle and demonstrated significant modulation of aminoacyl-tRNA biosynthesis pathways (Li et al., 2022). Correspondingly, in barley, Tandem Mass Tag (TMT)-based quantitative proteomic analysis was employed to characterize responses to aluminum stress under the interactive influence of phosphorus and *Piriformospora indica*. The resulting differentially expressed proteins (DEPs) were predominantly enriched in the phenylpropanoid biosynthesis pathway, with peroxidases emerging as the most prominent contributors. The combinatorial application of *P. indica* and phosphorus was found to enhance barley tolerance to aluminum-induced stress by modulating the antioxidative defence system (Feng et al., 2023).

Spatial proteomics is essential for understanding cell biology through protein localizations and subcellular dynamics. Moreover, with major advances in microscopy, mass spectrometry, and machine learning analytics, proteome-wide spatial studies are now achievable (Lundberg and Borner, 2019). Spatial proteomics surpasses methods like Cytotrap in numerous respects (Mohan et al., 2023; Yang et al., 2023), and advanced proteomics technologies, encompassing iTRAQ and label-free LC-MS/MS workflows, have progressively refined our ability to capture subtle yet consequential shifts in stress-related protein expression (Yang et al., 2023).

### 2.4 Metabolomics

Plant metabolomics is a rapidly advancing field bridging plant sciences and systems biology, which encompasses comprehensive analysis of small-molecule metabolites in plant tissues and cells, including sugars, amino acids, organic acids, secondary metabolites (alkaloids, flavonoids), lipids, and related compounds (Manickam et al., 2023). Current plant metabolomics employs high-throughput platforms, namely, gas chromatography-mass spectrometry (GC-MS), liquid chromatography-mass spectrometry (LC-MS), capillary electrophoresis-mass spectrometry (CE-MS), and nuclear magnetic resonance (NMR) spectroscopy, to enable robust detection, accurate quantitation, and comprehensive annotation of various metabolites (Kumar et al., 2024). Sophisticated computational algorithms and multivariate statistical methodologies, frequently integrated with machine learning approaches, are employed to discern critical metabolite signatures and stress-specific biomarkers, thereby facilitating targeted metabolic reprogramming strategies (Misra, 2021). Moreover, spatial metabolomics reveals tissue-specific metabolic signatures in stress responses, thereby revealing novel insights into localized adaptation mechanisms (Kim et al., 2024). Numerous metabolomic investigations have revealed substantial alterations in metabolic profiles under diverse biotic and abiotic stress conditions. For instance, metabolomic characterization of water-deficient rice revealed elevated levels of osmoprotectants, notably proline, trehalose, and raffinose coupled with altered concentrations of hormonal signaling molecules such as ABA and jasmonic acid (Gargallo-Garriga et al., 2018; Lu et al., 2023). In wheat, an integrated physiological and metabolomics analysis was conducted to characterize seedling responses to salt stress (150 mM NaCl) and heat stress (42 °C for 4 hours). Profiling of leaf primary metabolites revealed amino acids, sugars, and sugar derivatives as the principal discriminant metabolites under the imposed stress conditions. Integrated metabolomics analysis further identified ABC transporters, glucosinolate metabolism, aminoacyl-tRNA biosynthesis, cyanoamino acid metabolism, and galactose metabolism as the most significantly overrepresented pathways under the combined stress treatment (Shunkao et al., 2023).

### 2.5 Ionomics

The field of ionomics promises to deliver innovative frameworks that facilitate both a comprehensive understanding of ionome dynamics during environmental stress and the identification of genes and regulatory networks governing mineral accumulation, translocation, and participation in diverse molecular mechanisms under both homeostatic and stress conditions (Baxter, 2015; Ali et al., 2021). Multiplexed analytical platforms, particularly Inductively Coupled Plasma Mass Spectrometry (ICP-MS), facilitate the concurrent measurement of diverse elemental constituents across plant tissues (Huang and Salt, 2016). This discipline plays a crucial role in enhancing the climate resilience of crops. For instance, ionomic investigations have demonstrated that sulfur-based fertilizers ameliorate drought tolerance in maize by not only augmenting antioxidant biosynthesis but also enhancing photosynthetic efficiency, stomatal conductance, and transpiration rates (Usmani et al., 2020). Although numerous inorganic elements exhibit toxicity at elevated concentrations, mineral nutrients and trace elements play an indispensable role within safe thresholds in ameliorating the detrimental effects of abiotic stresses, including drought, salinity, and heavy metal toxicity (Singh et al., 2016). Among these, Ca²^+^ serves as a multifunctional secondary signaling messenger, participating in virtually all abiotic stress-mediated signaling cascades in plants. Exogenous application of Ca²^+^ has been demonstrated to effectively induce tolerance against a broad spectrum of abiotic stresses in crop species. Notably, the combined application of 24-epibrassinolide (EBL) and calcium was found to reverse salt-induced physiological alterations through the augmentation of ascorbate-glutathione cycle enzymes, antioxidant machinery including superoxide dismutase and catalase and osmoprotectant accumulation, encompassing proline and glycine betaine, collectively enabling tomato plants to withstand salt-induced toxicity (Ahmad et al., 2018).

## 3 Deep phenotyping platforms

Deep phenotyping platforms are sophisticated systems designed to quantify traits on a large scale, moving beyond traditional methods to accelerate research by employing advanced sensors and data collection systems (Li et al., 2021; Roitsch et al., 2019). These platforms enable non-destructive and high-throughput measurement of plant traits across development and environments, helping to capture temporal variation that is difficult to assess using conventional phenotyping methods (Cobb et al., 2013; Yang et al., 2020). Different types of platforms exist, such as high-throughput plant phenotyping platforms (HT3Ps) which monitor and evaluate plant phenotypes in environments ranging from greenhouses to open fields using methods like ground-based proximal phenotyping (growth chambers, hand held instruments, ground rovers) and aerial remote sensing (UAVs/Drones, Low earth orbit satellites) (Li et al., 2021). The sensors used in these systems are diverse; plant phenotyping often utilizes RGB, RGB-D, hyperspectral, thermal infrared, near-infrared cameras, NMR/MRI and PET as well as LiDAR and CT scanners (Li et al., 2020), with advancements also incorporating laser sensors and drones (Ninomiya, 2022). By integrating imaging sensors with automated data acquisition and analysis, these platforms allow quantitative assessment of plant growth, architecture, and physiological responses under controlled and field conditions (Fahlgren et al., 2015; Araus and Kefauver, 2018). Importantly, deep phenotyping supports the linkage of phenotypic variation with underlying genetic and molecular information, thereby strengthening genotype–phenotype analysis in crop improvement programs (Araus and Kefauver, 2018; Yang et al., 2020). The following subsections outline major imaging modalities used in deep phenotyping, highlighting their principles, applications, and relevance for capturing complementary dimensions of plant structure and stress responses.

### 3.1 RGB imaging

RGB imaging remains the most widely adopted modality in high-throughput plant phenotyping due to its low cost, ease of deployment, and direct link to visually meaningful traits. Standard RGB cameras capture visible-light reflectance to produce high-resolution images that yield quantitative descriptors such as projected leaf area, canopy cover, and greenness indices (Brichet et al., 2017; Lozano-Claros et al., 2020). These metrics are readily collected in both controlled environments and at plot scale in the field, supporting early-stage screening and large-scale breeding trials.

Effective RGB phenotyping relies on robust image processing pipelines to translate raw data into biological insights. The primary challenge is distinguishing plant tissue from complex backgrounds through segmentation and masking. Common approaches include color-space conversion followed by thresholding techniques like Otsu’s method (Brichet et al., 2017). More advanced workflows utilize machine learning frameworks, such as Ilastik or PlantCV, to implement decision trees or K-means clustering for robust foreground-background separation (Lozano-Claros et al., 2020). Automated segmentation can reach accuracies as high as 93% when using multi-step registration-classification approaches (Henke et al., 2020). To ensure data integrity, filtering and quality control steps often include histogram stretching for brightness adjustment and the application of median or Gaussian filters to remove salt-and-pepper noise (Lozano-Claros et al., 2020). QC steps may also involve down-scaling high-resolution images to optimize processing speed without losing critical morphological detail (Bethge et al., 2023). In longitudinal studies, temporal alignment is essential for tracking growth trajectories. “Two-step registration” techniques are used to align images taken at different time points, compensating for minor plant movements or camera shifts between imaging sessions (Henke et al., 2020). This enables the extraction of dynamic features like growth rates and developmental stage transitions.

Structure-from-Motion photogrammetry has become a cornerstone for analyzing canopy architecture. By processing overlapping RGB images, SfM workflows generate 3D point clouds, Digital Surface Models, and orthomosaics (Brocks and Bareth, 2016; Li et al., 2020). These outputs allow researchers to compute complex traits that are difficult to measure manually, such as convex hull volume, leaf angles, and canopy surface variability (Hrzich et al., 2025; Jay et al., 2014). While LiDAR provides high structural detail, SfM offers a more cost-effective and scalable alternative for estimating biomass proxies and plant height across large breeding populations (Hrzich et al., 2025; Li et al., 2020).

While often associated with aerial imaging, RGB is a primary modality for non-destructive root phenotyping. Using rhizotron or “clear-pot” systems, RGB cameras capture the “hidden half” of the plant by imaging roots as they grow against transparent surfaces (Alsalem et al., 2021; Narisetti et al., 2021). Unlike X-ray Computed Tomography or MRI, which are often limited by low throughput and high costs, RGB-based rhizobox imaging allows for the simultaneous monitoring of hundreds of individuals (Downie et al., 2014). While optical methods typically capture 20–85% of the total root system depending on the setup, they provide the high-frequency temporal data necessary to track root elongation and architecture dynamics in real-time (Alsalem et al., 2021; Downie et al., 2014).

The main limitations of RGB sensitivity to illumination, shadowing, and background variability are mitigated through standardized acquisition protocols and robust pipelines (Lozano-Claros et al., 2020). When these challenges are addressed, RGB imaging provides a high-value, scalable backbone for integrating structural and growth phenotypes with molecular data.

### 3.2 Spectral imaging

Spectral imaging expands the range of detectable plant traits by capturing reflectance information beyond the visible wavelengths, enabling the assessment of biochemical and physiological attributes that are not apparent in RGB images. Both multispectral and hyperspectral systems have been applied successfully to quantify stress responses, nutrient status and disease symptoms at leaf and canopy scales. Hyperspectral imaging, in particular, has been used to discriminate subtle physiological changes because fine-resolution spectral signatures are highly sensitive to pigment composition, water content and structural characteristics of plant tissues.

Early disease-focused studies demonstrated the utility of hyperspectral signals for detecting fungal infections in sugar beet leaves, where spectral differences enabled accurate separation of healthy and diseased tissue in controlled experiments (Mahlein et al., 2012). Likewise, Bohnenkamp et al. (2019) showed that spectral-decomposition approaches could differentiate between wheat brown rust and yellow rust, highlighting that disease-specific reflectance patterns can be isolated even when symptoms visually overlap. Building on this, Bohnenkamp et al. (2021) generated a time-series hyperspectral library of major wheat foliar diseases, providing a reference framework for disease classification across developmental stages.

Spectral imaging has also been applied to nutrient and biomass assessment. Zhang et al. (2024a) used hyperspectral imaging to estimate nitrogen content in soybean leaves under field and greenhouse conditions, reporting strong predictive performance using chemometric models. At the canopy scale, Yin et al. (2024) integrated UAV multispectral imagery with structural features to estimate aboveground biomass in summer maize, demonstrating the value of combining spectral indices with canopy-height information. Similar UAV-based multispectral approaches have been effective for biomass modeling in potato (Luo et al., 2022).

Despite its strengths, spectral imaging requires careful calibration, noise reduction and band-selection to avoid overfitting, particularly with high-dimensional hyperspectral data. Even so, the ability of spectral measurements to detect early physiological changes makes them a strong complement to morphological traits collected from RGB systems.

Multispectral systems are more cost-effective for agricultural monitoring than expensive hyperspectral systems, due to high sensor complexity and data-processing demands (Kontogiannatos et al., 2020). Hyperspectral offers superior spectral resolution for precise biochemical traits, nutrient status, and early stress detection (Galieni et al., 2021), effective for crop health monitoring (disease, nutrients, biomass) (Kontogiannatos et al., 2020). Challenges include complex calibration, high-dimensional data management, and model overfitting (Ahmad et al., 2024).

### 3.3 Thermal imaging

Thermal imaging quantifies leaf or canopy temperature by measuring long-wave infrared radiation, providing an indirect indicator of transpiration rate and stomatal activity (Boesch, 2017). Because evaporative cooling lowers leaf temperature, thermal measurements offer a practical approach to infer plant water status and detect stress responses before visual symptoms appear (Petrie et al., 2019). Several studies have demonstrated that thermal data can resolve fine-scale physiological variation in both controlled and field environments (Paz, 2021; Parra et al., 2021).

At the leaf scale, thermography has been used to derive stomatal conductance through energy-balance frameworks. Vialet-Chabrand and Lawson (2019) demonstrated that dynamic thermograms can be translated into stomatal conductance estimates with high temporal resolution, capturing rapid fluctuations in leaf behavior. Follow-up work validated thermography-based indicators of stomatal responses across changing environmental conditions (Vialet-Chabrand and Lawson, 2019). Similarly, Iseki and Olaleye (2020) proposed a temperature-derived index that correlates with measured stomatal conductance, offering a simplified method for detecting changes in leaf transpiration.

At the canopy scale, thermal imaging is widely used for water-stress detection and irrigation monitoring. UAV-based experiments have shown that thermal imagery can distinguish plots under varying irrigation regimes and map spatial patterns of canopy temperature linked to water deficit (Stutsel et al., 2021). Quantitative thermal indices, including the crop water stress index (CWSI), have been applied to diagnose drought severity in winter wheat, where elevated canopy temperatures correspond to reduced transpiration and stomatal closure (Ma et al., 2024). Integrated multisensor studies also highlight the value of combining thermal data with RGB or spectral imaging to improve detection of moisture deficits and soil–plant water relationships (Dong et al., 2024).

Thermal imaging has increasingly been incorporated into breeding pipelines. Field studies screening genotypes under deficit irrigation have shown that thermography can identify cooler-canopy genotypes that maintain higher transpiration and exhibit improved performance under water stress (El-Hendawy et al., 2024). Although thermal measurements are sensitive to environmental conditions such as wind, radiation load and humidity, standardized acquisition and calibration procedures enable reliable use of thermal traits for high-throughput phenotyping and climate-resilience breeding.

Thermal imaging systems are moderately priced and offer moderate spatial resolution, with strong applicability in water-stress detection, irrigation management, and drought-tolerance screening under field conditions (Yun et al., 2024). Their principal advantage lies in providing indirect yet rapid indicators of stomatal conductance and plant water status (Mayanja et al., 2024). However, measurements are highly sensitive to environmental fluctuations (radiation, wind, humidity), requiring careful standardization and calibration for reliable interpretation (Bayoumi et al., 2015).

### 3.4 Fluorescence imaging

Fluorescence imaging has proved a critical non-invasive method of developing climate resilience in crop plants through provision of multi-omics and deep phenotyping strategies. Abiotic stresses like drought and excessive temperatures are increasing with global climate change, and thus the creation of resistant crop varieties should be developed faster (Thingujam et al., 2025). Fluorescence methods, most eminently chlorophyll fluorescence imaging can give essential information on plant physiological system and photosynthetic performance, frequently recognizing stress effects before those situations emerge (Gorbe and Calatayud, 2012; Knopf et al., 2024; Wu et al., 2024). There has been a major breakthrough with the invention of Near-Infrared II fluorescence imaging. This method leads to a significant enhancement of the penetration depth and signal-to-noise ratio by suppressing tissue scattering and autofluorescence, allowing non-invasive imaging of the biological tissue at millimeters and even a single cell level with a molecular object (Schmidt et al., 2024; Wang et al., 2024). In addition to it, Angstromb-resolution fluorescence microscopy can now be used to resolve single-proteins in intact cell, so super-resolution microscopy meets structural biology again (Reinhardt et al., 2023). Moreover, the Fluorescence Lifetime Imaging Microscopy can provide distinctive functional information (Chang et al., 2007). The development of novel ChlF imaging techniques recently enables experiencing the full data of measuring photosynthesis and plant fitness even under stress. An example of this would be in handheld multifunctional fluorescence imagers; which quantitatively determine drought stress through measuring photosynthetic activity and quantification of biochemical outputs such as anthocyanins, demonstrating potential in high throughput phenotyping, both in controlled and field settings (Zhang et al., 2022). Spectral imaging also improves this by allowing differentiation of overlapping fluorophores and removing interference of fluorescence, making the signal more specific (Czymmek et al., 2025). Fluorescence phenotyping in combination with multi-omics (genomics, transcriptomics, proteomics, metabolomics, and epigenomics) play a vital role in understanding the intricate molecular pathways the plants use in their response to stress and hasten breeding (Amin et al., 2025a; Thingujam et al., 2025). Deep phenotyping and more particularly micro phenotyping bridges the gap between genotype and complex macroscopic phenotypes, and explains how genes and environments interact at a number of levels (Zhang et al., 2024a). ChlF-based thermal imaging types of instruments can continuously align dynamic variations in the space of leaf transpiration and photosynthetic performance under a stress condition and deliver specific indicators of stress diagnosis (Chaerle et al., 2009; Dwivedi et al., 2013). Recently, there is a recent demonstration of utilizing integrated multi-omics and advanced phenotyping to help underutilized crops such as *Camelina sativa* to create climate-smart varieties (Großkinsky et al., 2023). What is more, AI-powered microscopic 3D/4D phenotyping has become the future, as it can create large-scale, multidimensional plant micro phenotypes that would decode the complexity of genotype environment management (GxFx E Migration) interactions (Zhang et al., 2024a). These multi-scale systems using fluorescence imaging are necessary in creating climate resistant crops to secure food productivity in the world. Fluorescence imaging systems range from moderately priced handheld chlorophyll fluorometers to costly advanced setups (Sánchez‐Moreiras et al., 2020). They offer high physiological resolution for early stress detection, assessment of photosynthetic efficiency, and controlled-environment phenotyping, with strong pre-symptomatic sensitivity and functional correlations (Valcke, 2021). However, limitations include bulkiness, contact requirements, and operational challenges under field conditions, particularly signal saturation in complex canopies, deeper leaf layers, or under daytime illumination (Zhang et al., 2022).

### 3.5 IR imaging (NIR, FTIR)

Non-destructive and label-free chemical characterization in the form of infrared imaging, which includes Near-Infrared (NIR) and Fourier Transform Infrared (FTIR) spectroscopy, is the most powerful tool that has catalyzed major changes in the various fields of science. The methods offer spatially resolved molecular data, which is essential to the study of complex systems ranging between biological tissues and more intricate materials (Lan, 2021; Pierna et al., 2020; Stegemann et al., 2024).

#### 3.5.1 Near-infrared imaging

Near-Infrared Imaging is used to visualize tissue optical properties (including absorption and scattering) and the shift of surface temperatures by monitoring alterations in temperature or concentration on the body’s surface as part of nonionizing radiation. NIR imaging is becoming increasingly popular because it can measure deeper tissue characteristics and reduced scattering, which characterize tissue surfaces of the investigated animal (Wang et al., 2024). NIR spectroscopy is highly efficient in detecting biochemical variations in plants, determining parameters such as water content and seed quality, thereby simplifying the high-throughput phenotyping of biotic and abiotic stresses (Gouveia et al., 2020). In particular, high-performance FT-NIR is capable of providing a better spectral resolution and signal-to-noise ratio, which increases the accuracy of these measurements (Visakh et al., 2024). Recent researches prove the opportunities of NIR to measure plant biomass and water status during drought stress, such as in rice (Kim et al., 2020). NIR spectroscopy can be used as a rapid, precise, and accurate tool to measure multiple characteristics associated with plant morphology, chemistry, and metabolism due to its integration with deep learning algorithms, which makes phenomic studies much faster (Vasseur et al., 2022).

#### 3.5.2 Fourier transform infrared imaging

Alternatively, FTIR spectroscopy is invaluable due to the potential of estimating the metabolites as well as an in-depth information regarding the chemical structure of the plants and reaction to ecological stimuli. FTIR is clear in terms of data quantification, which gives biochemical changes at cellular and molecular scales that are essential in predicting stress tolerance. As an illustration, FTIR has been used to investigate the stress of potassium in cotton, clarify the effect on the cell wall pectin, membrane permeability, protein status, and the quantity of polysaccharides (Visakh et al., 2024). FTIR spectroscopy has been used successfully to estimate cold acclimation-induced metabolic changes at the cellular and tissue levels. Furthermore, synchrotron-based FTIR analysis revealed significant differences in cell wall composition in Fusarium-infected wheat, offering high-resolution insights into pathogen-induced structural and chemical changes.

NIR/FTIR synergy changes in combination with other sophisticated imaging tools allow deep phenotyping of linking minor changes in molecules to palpable phenotypes of the plant. Hyperspectral Imaging also incorporates NIR and typically FTIR crowds, so it is valuable in early detection of abiotic stresses such as drought and toxicity to heavy metals, making it useful in crop species such as maize (Muhammad et al., 2025). Such techniques are able to yield holistic data that can be combined with multi-omics data (genomics, transcriptomics, metabolomics) to reveal the underlying complex regulations that lead to plant adaptation and resilience. Such multi-scale data merge is necessary to build powerful predictive models and breeding methods based on climate-smart agriculture (Juárez and Kurouski, 2024; Thingujam et al., 2025).

NIR systems are moderate/low-cost and suited for high-throughput biochemical estimation (water content, biomass, seed quality) (Vasseur et al., 2022). FT-IR is costlier and lab-oriented, needing facilities/training/LN2 cooling (mid-IR > vis-NIR) (Whatley et al., 2023). Both offer molecular specificity/non-destructive profiling (Zhang et al., 2023), but require robust calibration for overlapping bands (Bharadwaj et al., 2024) and suit field less than RGB/multispectral (miniaturized NIR recent) (Yan et al., 2019). To sum up, IR imaging method, in theme NIR and FTIR, is irreplaceable with the tools of non-destructively measuring the health of plants, their biochemical structure and reaction to environmental stimuli. They are also central to filling the gap in genotype to phenotype research and boosting the production of climate resilient crop varieties to ensure global food security, in part because of their ability to generate high-resolution, multi-scale data, particularly in conjunction with more sophisticated analytics and multi-omics methods.

### 3.6 Tomography imaging

Tomography imaging, which includes Nuclear Magnetic Resonance/Magnetic Resonance Imaging (NMR/MRI), Positron Emission Tomography (PET), and X-Ray Computed Tomography, play a central role in scaling the bridging and integrating multi-omics data and promoting climate resilience in crop plants (Galieni et al., 2021; Harandi et al., 2023; Ye et al., 2023). Such non-invasive techniques provide more information on how plants react to environmental stress than ever before, which is essential at a time when global food security is facing rapid changes and growing demands (Bäurle et al., 2022; Mansoor and Chung, 2024; Nguyen et al., 2025). These technologies allow studying the interactions between the genotype and the environment and management more thoroughly and comprehensively, using three-dimensional representations and studying dynamic processes within the organism, which leads to easier and faster creation of stress-resistant plant types.

The magnetic resonance imaging (MRI) makes it possible to measure plant structures and physiological processes in a non-destructive and three-dimensional format, and it is highly sensitive to the level of water content and water transportation (Blystone et al., 2024). It offers detailed visualization of the internal root and shoot structure and enables the specific mapping of xylem and phloem-related water transport that is essential in understanding the hydraulic behavior of plants and their drought behavior. The recent research evidence has been applied in the measurement of internal defects in the potato tubers with the use of MRI due to water stress to determine the water stress levels and a means of the moisture level in the kernels in maize, which is useful in an accurate phenotypic screening of drought tolerance in breeding programs (Angidi et al., 2025). MRI has been used to determine the change in cellular and tissue level water allocation in leaves of Brassica napus when in a dehydration state (Cai et al., 2025). Other than the abiotic stress measurement, MRI enables the early diagnosis and the locution of plant diseases such as Verticillium wilt in cotton and infection of strawberry seedlings by *Phytophthora cactorum* (Cai et al., 2025; Tuomainen et al., 2024). Its non-invasiveness allows making measurements of the same subject several times, and longitudinal studies of growth, stress development, and recovery can be performed. These are especially useful in decomposing phenes related to complex phenes in terms of water use efficiency, as well as stress resistance. New portable MRI systems continue to expand these uses by allowing *in situ* measurement of plant water status and root structure-function associations in the natural environment (Nuixe et al., 2024).

Positron emission tomography (PET) uses radioactive tracers with short half-lives to image the metabolites and non-invasive transport of photo assimilates, nutrient and phytohormones in plants (Galieni et al., 2021; Pisante et al., 2022; Ruwanpathirana et al., 2021). This functional imaging is able to get rare data on real-time carbon assimilation and allocation under stress, therefore, clarifying carbon partitioning options that are directly involved in plant growth and yield (Streun et al., 2024). Compartmental modelling of phloem and xylem transport (quantitative PET) has also demonstrated the possibility of early identification of the adverse effects of stress due to climate change (Antonecchia et al., 2022). Secondly, the dynamics of sodium transport in barley have been tracked by PET which offers mechanistic information on the uptake of ions during nutrient deprivation and under ion channel inhibition, the two fundamental mechanisms of salinity tolerance (Ruwanpathirana et al., 2021). Together, dynamic imaging with PET can provide an effective paradigm to decipher physiological reactions of plants to environmental changes and the most effective use of nutrients in crops (Mincke et al., 2021).

X-ray computed tomography (CT) with micro-CT produces high-resolution in three dimensions of internal plant tissues and root systems (Harandi et al., 2023). It is an effective instrument of non-destructive root phenotyping, which allows the direct quantification of the root system architecture characteristics of root length, diameter, volume, and branching in soil (Liu et al., 2020; Herrero-Huerta et al., 2022). Utilizing the natural soil-root environment, X-ray CT provides the key information on the water and nutrient exploration processes of the roots in the soil, which serves as the basis of plant health in the stressful environment (Harsselaar et al., 2021; Ghosh et al., 2023). The method assists in spatio-temporal observation of root development and demonstrates the existence of adaptive architectural plasticity to abiotic and biotic stressors (Ghosh et al., 2023). In addition to root systems, X-ray CT has been used in characterizing complex reproductive and yield-related geometries, such as grain setting and grain size in wheat ears classified under drought and heat stresses, which reveal the effect of stress on structural alterations in grains (Schmidt et al., 2020; Langstroff et al., 2021). The reconstructions 3D also allows the precise assessment of such morphological parameters as tissue volume and surface area, which can be utilized in the breeding of precision breeding methods (Gao et al., 2025). The assessment of root architectural plasticity has also been applied to produce stress-tolerant crops like rice (Teramoto et al., 2020; Teramoto and Uga, 2022) with the help of X-ray CT and to measure three-dimensional seed architecture and cellular features, as evidenced in pennycress (Griffiths et al., 2025). Using CT and combining it with complementary methods like scanning electron microscopy improves the use of multi-scale and cellular-level visualization of plant structure and functioning (Zhang et al., 2024a).

It is critical that these tomographic techniques be further converged with multi-omics and deep phenotyping so as to understand the thoroughly genotype environment-management interactions. These imaging modalities, along with high-throughput phenotyping technologies, cannot be done without because they are critical in the measurement of essential characteristics of large populations in a non-destructive and high-throughput manner (Angidi et al., 2025). The ability of researchers to produce multidimensional and large-scale data sets of all organizational levels of the plant would allow one to accurately assess intricate characteristics and determine the genetic regulatory interventions of stress resilience (Sahoo et al., 2020). This interdisciplinary model leads to the evolution of climate-sensitive agriculture whereby selection of stress-resistant crop varieties becomes possible, which is critical in global food security innovations will probably imply AI-enabled 3D/4D imaging and analysis system, wherein machine learning and deep learning are paired (Yang et al., 2023). Calculation strategies will increase the precision of the phenotypic study and improve the speed at which the breeding procedure works, eventually leading to a much more sustainable and resilient agricultural future (Khan et al., 2022). However, tomographic techniques are the most expensive imaging modalities, with high costs and infrastructure demands limiting broad availability, especially for micro-CT. They offer very high spatial and functional resolution for non-destructive 3D visualization of internal structures and processes, suiting root phenotyping, tissue analysis, carbon allocation, and stress research (Rahaman et al., 2015). Yet, low throughput from reconstruction/classification bottlenecks and technical complexity constrains routine breeding use (Gomez et al., 2018; Roose et al., 2016).

### 3.7 Other 3D imaging

Further non-invasive techniques, especially LiDAR and other high-throughput phenotyping systems are transforming our capacity to comprehensively phenotype the crops and then combine such results with multi-omics measures. Non-destructive and precise to obtain large-scale phenotypic data, high-throughput phenotyping is relevant as a solution to essential bottlenecks when employing traditional breeding and genomic research approaches (Kim et al., 2020, Kim et al., 2021). Light Detection and Ranging is one of them due to its ability to create accurate three-dimensional (3D) structural data of the vegetation and canopies (Jin et al., 2020). Even in conditions of different drought stress, Unmanned Aerial Vehicle-based LiDAR systems have proven useful in high-throughput determination of plant height and overground biomass extending to provide valuable information used in trait analysis (Maesano et al., 2020). LiDAR combination with other remote sensing modalities, including hyperspectral, multispectral, and thermal imaging, develops highly powerful multi-sensor schemes to monitor the health of plants in detail and analyze their genetic traits (Zheng et al., 2021). A recent research is an illustration of the strength of such combined strategies. As an example, multi-scale remote-sensing phenomics, when combined with multi-omics data (genomics, transcriptomics, metabolomics), is expanding the current knowledge of the mechanism of crop drought-heat stress tolerance (Liang et al., 2025). AI and machine learning can be used to speed up breeding programs because such integration allows the correct prediction of traits related to stress tolerance and the optimization of selection modes (Amin et al., 2025a; Roth et al., 2025). These approaches play an essential role in defining stress-resistant genotypes as well as tracking plant stress to different environmental issues such as drought, salinity and extreme temperatures (Angidi et al., 2025; Nguyen et al., 2025). These characteristics of capturing the finely functional phenotype, including dynamic responses to water scarcity are strategic in the development of drought-tolerant cultivars, water resource management (Mansoor and Chung, 2024). Regardless of such an impressive advancement, there are still issues to build cost-effective, high spatial-temporal, and hyperspectral LiDAR impeccable plants, as well as new algorithms to develop multi-dimensional phenotyping (Jin et al., 2020). However, unremitting advancement of such imaging technologies, in conjunction with advanced data analytics, makes them a necessity in bridging the gap between biology and speeding up the creation of climate-resistant crop plants to ensure sustainable agriculture. However, despite these advances, LiDAR systems range from moderate to high cost depending on platform integration (Toit et al., 2022) and deliver high spatial 3D structural resolution at canopy scale (Jin et al., 2020). They suit field plant height estimation, biomass modelling, and structural trait analysis (Jiménez-Berni et al., 2018), enabling precise 3D canopy reconstruction and multi-sensor integration, but lack biochemical data, involve complex point cloud processing, and limit small-scale breeding due to cost (Walter et al., 2019; Yang et al., 2017).

## 4 Strategies for integration of multi-omics and deep phenotyping

### 4.1 Association mapping

The building block of genome discovery has been the association mapping and specifically Genome-Wide Association Studies method, which has been used to dissect the genetic architecture of climate resilience (Bhusal et al., 2023). GWAS can be used to identify the particular genomic area, quantitative trait loci, and candidate genes that are linked to the attributes that include drought, heat, or salinity tolerance by taking advantage of natural genetic variation among various crops (Wang and Qin, 2017; Mosalam et al., 2025). The method of genotyping on high-throughput scale has made it possible to cover large regions of markers and, as a result, allow GWAS to identify new alleles more quickly, despite the fact that the method has generally limited itself to the bi-parental level of mapping population (Kole et al., 2015; Slawin et al., 2024; Badr et al., 2025). Climatic resilience is a complex property and the multi-omics technologies can present a global account of response of plant to environmental stressors at both a molecular scale. After establishing genomic regions of interest that have been identified by association mapping, multi-omics techniques provide a further insight into their functional setting (Syeda, 2025; Thingujam et al., 2025).

Traditional phenotyping has been a traditional bottleneck in breeding of complex traits. Phenomics or deep phenotyping is a solution to this using high-throughput, non-destructive and accurate measurements of physiological and morphological phenotypes and morphology in controlled and field settings (Chang-Brahim et al., 2024). Remote sensing technologies, use of remotely operated drones and hyperspectral systems allow real-time observation of the performance of plants, with sensitive and dynamic behavioral changes including alterations in plant structure, the effectiveness of photosynthesis, and water utilization (Liang et al., 2025).

This is important phenotypic data that can be used to link genetic data through association mapping to molecular information through multi-omics and plant performance as well as enhance the breeding cycle (Thingujam et al., 2025). Computational requirements associated with the integration of both massive, multi-dimensional datasets produced by association mapping, multi-omics, and deep phenotyping are very large. Deep learning, and the wider approaches of AI and Machine learning framework, is the key due to the ability to process, analyze, and interpret these complex data streams (Wang et al., 2020; Zhu et al., 2024). It has been shown that deep learning models can also help uncover complex patterns, can make accurate predictions and optimize breeding strategies through bridging the gap between genotypes and phenotypes (Li et al., 2025). They examine the relationship between genetic changes (capability of GWAS) and molecular changes (capability of multi-omics) and their subsequent expression to produce observable characteristics (phenotypes) to accelerate the process of identifying better germplasm and the creation of climate- resilient breeds.

The recent crop-specific research may clearly show the power of enforcing genomics, multi-omics, deep phenotyping, and AI to increase the resilience to climate changes. Genome-wide association studies in wheat have found critical loci and new alleles associated with drought and heat tolerance (Bennani et al., 2022; Mosalam et al., 2025), and deep learning models such as DeepWheat combining genomic and epigenomic with phenomic data have improved significantly predictive capabilities of grain yield and gene regulation in case of stress (Ma et al., 2025). Association mapping in barley has discovered genetic determinants of drought, waterlogging and salinity tolerance at distinct developmental phases, and environmental genomic selection has assisted in identifying a landrace which would tolerate unpredictable and dynamic climatic conditions (Slawin et al., 2024; Badr et al., 2025). Integrated GWAS and deep learning methods have discovered genomic locations and candidate genes that regulate the heat and salinity tolerance in maize, and multi-trait multi-environment prediction models have proven more accurate to depict genotype-environment interaction on complex traits like flowering time (Mora et al., 2023; Montesinos-López et al., 2025). GWAS and QTL mapping of rice have also found positive alleles and candidate genes of heat tolerance, especially in high-night temperatures and during sensitive flowering periods (Bheemanahalli et al., 2021; Li et al., 2023). Here and further, multi-omics applications to soybean and potato research demonstrate that multi-omics combined with AI-based analytics and deep phenotyping could be used to discover more information about the role of genes in the underpinning of stress tolerance, nutritional quality, and adaptation in the presence of discrete and combined abiotic stress (Zhu et al., 2024; Zagorščak et al., 2024; Gai et al., 2025). These examples combined are showing how integrated, data-driven systems are speeding up the process of trait discovery, as well as enhancing breeding efficiency in an array of crop species. Combination of association mapping, multi-omics and deep learning often undergoes synergistic integration that is a paradigm shift in crop science. This multi-facet strategy offers a sound architecture with which to deconstruct the multifaceted characteristics, comprehend how plants respond to stress at the biological spectrum and hasten the creation and commercialization of climate-adaptive plant types. This paradigm cannot be done without in ensuring a sustainable and productive agricultural future.

### 4.2 Machine learning integration

**Figure 2:**
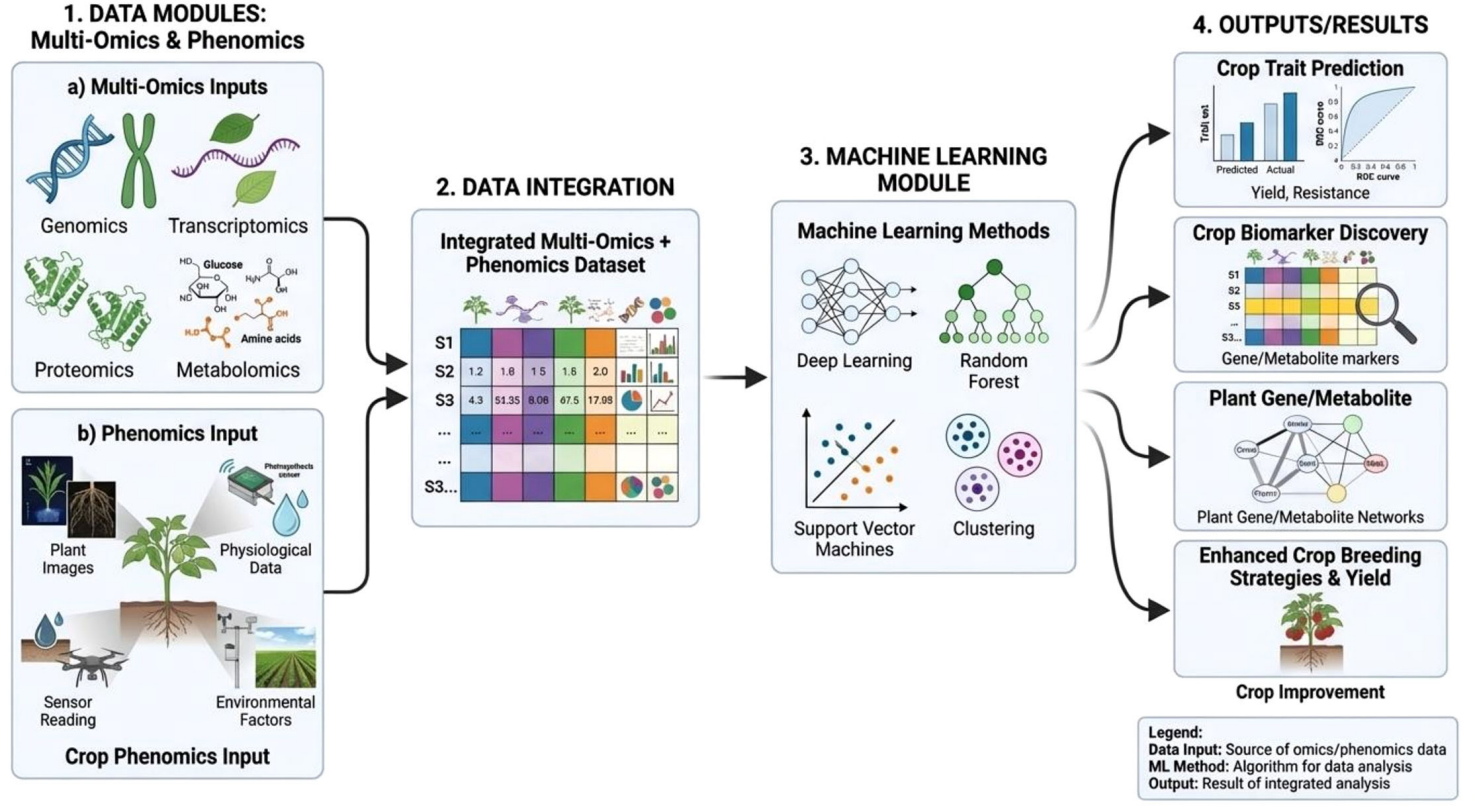
Basic steps involved in analyzing multi-omics and phenomics datasets using machine learning.

Integration of machine learning (ML) with high throughput phenotyping is a potential technological combination that can be used to overpower the consequences of climate change affecting crop improvement. The integration of multi-omics data (genomics, transcriptomics, proteomics, and metabolomics) with phenomics information to generate a comprehensive dataset for crop analysis. Machine learning approaches such as deep learning, random forest, and support vector machines analyze this integrated data to predict crop traits, identify biomarkers, and support advanced breeding strategies for crop improvement (**Figure 2**). Even though the efforts are in budding stage, such integrations have proved to be very much rewarding. To overcome the issue of prediction accuracy in case of complex data analysis using statistical software relaying of theoretical concepts and liners regressions, ML and AI (AI) can be a feasible solution (Esposito et al., 2019; Angidi et al., 2025). Integration of rich multiomics datasets with phenomics can unravel more conceptual and multi-disciplinary data-driven capabilities. In many crops like wheat, maize, soyabean etc., machine learning and deep learning algorithms trained with phenotypic imaging data could effectively predict crop yield and quality (Sandhu et al., 2021; Barbosa dos Santos et al., 2022; Wu et al., 2024). Such correlated outputs can be used as a reliable indicator in crop selection in crop improvement research. With the advancements in AI, multiple predictions algorithm based models have been experimented in multiple crops. Machine learning is classified based on how the algorithms learn from data and predicts a solution or conclusion. Based on this learning mechanism, a detailed categorization of machine learning methods and their utility is discussed below in detail.

#### 4.2.1 Supervised learning methods

Supervised learning is an efficient strategy in machine learning where labelled datasets are trained in such a way that each input feature has a corresponding output pair, enabling precise prediction and easy classification of prediction outcomes. This approach excels in regimes that require mapping of input to output tracking such as integrated phenotypic trait predictions based on multi-omics data. Mainly used supervised machine learning algorithms include regression algorithms; classification algorithms such as logistic regression and native bayes algorithm; and combined algorithms such as decision tree, random forest (RF), support vector machine (SVM), K-Nearest neighbors, gradient boosting algorithm, etc. Boosting algorithms such as Extreme Gradient Boosting (XGBoost) builds sequential trees to minimize prediction errors while processing high dimensional multi-omics data and phenotypic datasets (Amin et al., 2025a). Gaussian Process Regression models have the ability to handle uncertainty and are effective if the data is complex and highly overlapping. SVM models are generally help in mapping complex data into higher-dimensional space using kernels to classify or regress traits (Amin et al., 2025a).

**Figure 3:**
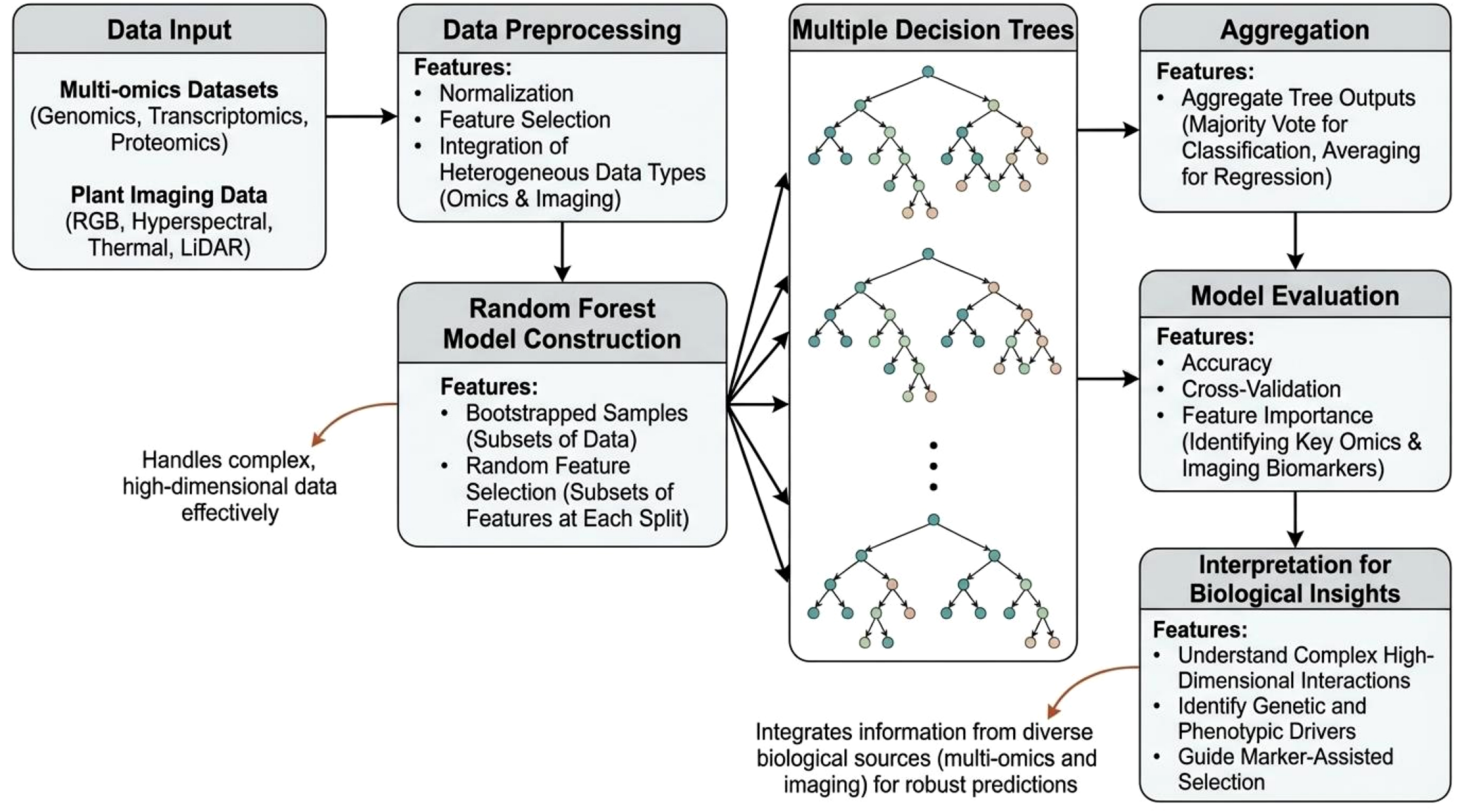
A flowchart on working of the random forest (RF) machine learning model for handling multi-omics and plant phenotyping datasets.

Among the supervised models, RF is more predominant due to its ability to deal missing values, high dimensionality, robustness, and interpretability (Jeong et al., 2016; Morabito et al., 2025). RF is an ensemble of decision trees which is capable of handling heterogeneous data well and captures non-linear interactions without intensive feature scaling. RF workflow start with the construction of an ensemble of multiple independent decision trees in the model development phase. Each of this trees are built using a random subset of training data and a random selection of input features. This diversity is instrumental in preventing overfitting. Later in the prediction part the model performs aggregation or majority voting technique from all trees, while regression averages the outputs. Model evaluation typically uses the cross-validation, followed by generation of biological insights based on scores identifying which specific factors affects the crop to the maximum extend. A flowchart on the various features and working of the random forest machine learning model, with focus on handling multi-omics and plant imaging data is illustrated in **Figure 3**. In maize crop, non-linear feature selection method based RF prediction model could effectively integrate SNP datasets with outputs from automatic phenotyping platforms for predicting crop yield (Wu et al., 2024). RF outperformed multiple linear regression-based models ((Partial Least Squares (PLS), Stepwise Regression (SR), Least Absolute Shrinkage and Selection Operator regression (LASSO), rrBLUP, Support Vector Machines with Linear kernel (SVR) and Elastic Net (EN)), neural network-based models (artificial neural networks (ANN), Gaussian process with radial basis function kernel (GaussprRadial) and deep neural network for genomic Prediction (DNNGP)) and other tree based models (Bayesian Additive Regression Trees (BART), Light Gradient Boosting Machine (LightGBM), Generalized Boosted Regression Models (GBM) and Multivariate Adaptive Regression Splines (MARS)). Superiority of RF over SVM, ANN and linear models were also reported in soyabean (Barbosa dos Santos et al., 2022). RF algorithms also works effectively with hyperspectral imaging data even in prediction of asymptomatic stress in plants (Poona et al., 2016).

#### 4.2.2 Deep learning architectures

Deep learning is an efficient means to handle very large and complex and often unstructured datasets through automated learning of hierarchical representations without any manual feature engineering. Deep learning architectures mainly include neural networks such as ANN, Convolutional Neural Networks (CNNs), Recurrent Neural Networks (RNNs)/LSTM and Graph Neural Networks (GNNs). These non-linear models are superior to linear models as they can provide better estimates of additive effects. Multi-layered deep neural network architecture has multiple layers constructed during model training. ANN models are generally preferred in case of numerical datasets with dense layers (Cembrowska-Lech et al., 2023).

CNN models were widely used in prediction of crop yield by training with hyperspectral and remote sensing data. Integration of Gaussian process component to the CNN model along with dimensionality reduction technique can help in explicit modelling of spatio-temporal data structure and in enhancing the learning capability even with scarce data (You et al., 2017). CNN also performs well in case of UAV or hyperspectral image feature integrated genomic data sequence motifs analysis (Thingujam et al., 2025). In case of time-series multi-omics or continuous phenotypic monitoring data with sequential dependencies such as growth curves, RNN models can be chosen over other neural networks (Theodorakis et al., 2024). Maintaining uniformity in imaging conditions in field is yet another area of concern in phenotyping which can have effect on prediction accuracies when integrated with omics data. In such cases, Deep neural networks (DNN) could efficiently predict crop yield across thousands of environments over the years compared to shallow neural networks (SNN), Lasso and regression tree (RT) (Khaki and Wang, 2019). Graph-based Neural Networks is another novel model that represent biological entities as nodes and multi-omics interaction as edges. It integrates diverse omics modalities in a network context, capturing interaction patterns that traditional models might miss (Theodorakis et al., 2024).

Studies indicate that, for prediction of phenotypes based on multi-omics data, a deep neural network model named neural network genomic prediction (DNNGP) developed for genomic prediction could provide more accurate predictions compared to GBLUP, light gradient boosting machine (LightGBM), SVR, and two other deep learning frameworks, namely deep learning genome-wide association study (DLGWAS) and deep learning genomic selection (DeepGS) (Wang et al, 2023). DNNGP with a multilayer hierarchical function for dynamic learning feature could provide faster and accurate prediction even small data sets. Even though models like DNNGP claims substantial improvement, majority of deep learning models fall short in grasping intricate patterns and representations when trained with small volume of sample data (Dou et al., 2023).

#### 4.2.3 Unsupervised methods

Unsupervised learning algorithm strategy learns, analyses and models the input data without any priority labelled responses or categories. Here the algorithm solely uses the input datasets to discover unidentified interaction patterns and relations without any prior knowledge. This method does not include data training stage. Mainly used unsupervised machine learning algorithms include clustering algorithms, associate rule learning and dimensionality reduction. Clustering algorithms generally help in forming multiple clustering integrations. Multiple omics data integration have resulted in a higher silhouette score, indicating better clustering quality due to better fitting of sample in the assigned cluster (Chong et al., 2022). Even with limited studies on clustering algorithms few models such as Deep Embedded Clustering (DEC) is capable in learning feature representation and developing cluster assignments with specific objectives (Xie et al., 2016). Unsupervised integration techniques such as PINSPlus empowered with underlying NEMO (NEighborhood based Multi-Omics clustering) algorithmic framework is capable of handling data without any need of interpolation and integrate these datasets by constructing similarity matrices across omics and clustering them for discovery of new phenotype or biomarker associations (Nguyen et al., 2019; Rappoport and Shamir, 2019).

#### 4.2.3 Kernel and hybrid methods

Kernel methods are ML techniques that transform complex, heterogeneous omics data into a similarity (kernel) space where relationships become easier to model without explicit feature engineering. Instead of working directly with raw features, kernel functions compute pairwise similarity between samples in a high-dimensional feature space. Hybrid approaches combine kernel methods with other machine learning or deep learning techniques to exploit the strengths of both frameworks. Kernel-based and hybrid approaches are tailored for handling heterogeneous multi omics datasets. For example in Multiple Kernel Learning (MKL) each data modality from multiple omics approaches gets its own kernel (a similarity measure) and these kernels are analyzed and combined to develop a predictive model (Briscik et al., 2024). MKL was reported to be an efficient, fast and reliable solution which outperformed various other complex architectures.

#### 4.2.4 AutoML and ensembles

Combination of machine learning models or ensemble methods is another approach that is currently used as an effective approach in booting the prediction accuracy. Here outputs of different models are used as inputs in a meta-learner module, which generally improves the accuracy of outcomes (Morabito et al., 2025). Multimodal deep learning such as AutoGluon integrates these diverse multi-omics and phenotyping data inputs into a single model that learns joint representations enabling richer phenotype predictions (Bai et al., 2024; Mohamedikbal et al., 2025). So, integration of machine learning in multi-omics and phenotyping data driven research can play vital role in dimensionality reduction, assessment of temporal and image data and also in capturing nonlinear and higher order interactions that most linear models and statistical tools fail to unravel. A table comparing the strength of multiple machine learning methods in multi-omics and phenotyping integrations studies are shown in **Table 1**.

**Table 1:** Machine learning methods used in high-throughput phenotyping integrated multi-omics studies in crops.

Sr. No.	Category of ML	Models	Advantages	Disadvantages
1.	Supervised	RF, SVM, XGBoost, PLS	Robust predictions, interpretable features; enabling direct mapping from multi-omics to target traits	Need of well labelled large data, hard to interpret
2.	Deep Learning	CNN, RNN/LSTM, Autoencoders, GNN	Learns complex non-linear patterns, integrates diverse data types and captures higher-order interactions	Demands big data and compute, overfitting, hard-to-interpret.
3.	Unsupervised	PCA, MOFA, clustering	Reveals hidden structure, reduces dimension without need of labels, aiding hypothesis generation and stratification	Sensitive to heterogeneity and missingness; clusters/factors are hard to validate
4.	Kernel/Hybrid	MKL, ensemble models	More flexible with combinatorial advantage of different algorithms; integrates heterogeneous omics	Scales poorly with high dimensionality, require delicate kernel/tuning across heterogeneous omics; difficult to interpret
5.	AutoML/ Ensembles	AutoGluon, stacked models	Automates model selection and tuning with hyperparameter selection, which often improves accuracy and robustness.	Computationally heavy; reduces transparency about which omics/features drive results.

### 4.3 Cross-validation schemes for omics and multi-omics models

In the context of crop improvement and plant breeding, cross-validation (CV) schemes are designed to simulate the real-world challenges of predicting how a specific plant genotype will perform in a specific field. Cross validation verifies the predictive ability of the model chosen for the analysis. The standard approach that is most commonly used is random CV where individuals are randomly assigned to folds regardless of their pedigree and environmental parameters. This model proves to estimate the general prediction ability within a specific population while the method often leads to over optimistic results. For specific populations mainly in agriculture experiments following Randomized Complete Block Design (RCBD) Blocked CV proves to be more effective as it ensures that each experimental block is kept together in the same field. If the experiment includes datasets from multiple environments, then Environment-wise CV approach which specifically targets genotype-environment interactions con be more rewarding (Jarquín et al., 2014). This approach can be specifically used in plant breeding experiments to predict the performance of a genotype in a new environment. Similarly time based approaches simulates next generation breeding cycle by accounting the climatic variations in the past (Tomar et al., 2021). In case of advanced plant breeding techniques like genomic selection aimed at plant selection, genotype-wise CV moving of all data points of a genotype to the test set and splitting by unique plant varieties, instead of splitting by samples is preferred (Mróz et al., 2024). This helps in predicting the performance of a completely new variety that hasn’t been phenotyped yet. Another modified approach that can be used is family-wise CV which ensures that an entire family is excluded from the training set, thereby helping in predicting performance in a distantly related genetic background. If it is observed that the prediction accuracy drops while using this scheme, that indicate that over reliance of the model on close pedigree relationships rather than the underlying biological datasets or markers. In case of multi- omics integrations, these CV schemes become more complex. When a horizontal integration CV is used, it ensures that for a given genotype-wise split, all omics layers for that genotype are removed simultaneously to prevent leakage. In case of trait-specific CV approaches, since different omics layers are more sensitive to the environment than molecular markers, environment-wise CV is mandatory to ensure the omics signatures are not just capturing noise from a specific field.

## 5 Applications of integrated strategies in crop improvement for climate resilience

Strategies integrating multi-omics and deep phenotyping provide transformative approaches for developing climate resilient crops with increased efficiency and precision. The approach involves combining genomics, transcriptomics, proteomics, metabolomics, phenomics and ionomics, which enables unprecedented insights into crop stress tolerance mechanisms (Yang et al., 2021; Amin et al., 2025a; Thingujam et al., 2025) with high-throughput phenotyping platforms such as drones, hyperspectral imaging and satellite sensors, which allow non-invasive assessment of stress indicators, while AI and machine learning algorithms analyze complex datasets to predict adaptive traits (Liang et al., 2025). For instance, GWAS combined with metabolite profiling techniques (mGWAS) has been used for elucidating genetic determinants of metabolic traits to provide molecular markers for breeding stress resilient crops (Thingujam et al., 2025) and these methods are proven to be effective in dissecting the biochemical and genetic processes in several model crop species including rice, maize and tomato (Luo, 2015; Matsuda et al., 2015).

### 5.1 Integrated strategies for abiotic stress resilience

Abiotic stresses that affect crop productivity under climate change include drought, extreme temperatures, salinity and waterlogging, which act across molecular and physiological scales. The simultaneous characterization of regulatory networks and dynamic stress responsive traits is made possible by the integration of multi-omics approaches with deep phenotyping platforms. This convergence facilitates the development of predictive breeding techniques for climate resilient crops by bridging genotype and phenotype (**Table 2**).

**Table 2:** Applications of integrated omics and phenotyping strategies for improving abiotic stress resilience in major crops.

Crop	Omics layer(s)	Phenotyping level	Key traits analyzed	Integration strategy	Application in crop improvement	Reference
Rice (*Oryza sativa* L.)	Genomics (GWAS using 6K genic SNP array)	Field based phenotyping	Yield per plant, number of productive tillers, spikelet fertility, number of filled grains, Na^+^/K^+^ ratio and stress susceptibility indices	GWAS using mixed linear models (K + P/K + Q)	Marker-assisted selection and pyramiding of salinity tolerance alleles in rice	Kumar et al. (2015)
Durum wheat (*Triticum turgidum* L. ssp. *durum*)	Genomics (GWAS; DArTseq SNPs)	Field based physiological and agronomic phenotyping	Grain yield, grain number, thousand grain weight, NDVI, phenology traits, stress tolerance indices (SSI, TOL, STI)	Multi environment GWAS integrating stress indices and trait BLUPs	Marker-assisted breeding for drought and heat resilient wheat cultivars	Sukumaran et al. (2018)
Cotton (*Gossypium hirsutum* L.)	Genomics (GWAS; whole-genome resequencing SNPs)	Physiological phenotyping (controlled conditions)	Germination rate, fresh weight, seedling length, relative water content, chlorophyll content, MDA and electrical conductivity	GWAS integrating phenotypic ratios (stress/control) with mixed linear models (EMMAX) and LD based candidate gene mining	Marker-assisted selection for salinity tolerance in cotton	Yasir et al. (2019)
Rice (*Oryza sativa* L.)	Genomics (GWAS using SSR markers)	Physiological and agronomic phenotyping	Na^+^, K^+^, Ca²^+^, Mg²^+^ content in leaf and stem, Na^+^/K^+^ ratios, chlorophyll content, grain yield and salt injury score	GWAS using mixed linear model (Q + K) and LD-based candidate gene identification	Marker-assisted selection and identification of donor genotypes for improving salinity tolerance in rice	Warraich et al. (2020)
Wheat (*Triticum aestivum* L.)	Genomics (GWAS; 35K SNP array)	High-throughput physiological phenotyping across multi location trials	NDVI, canopy temperature, chlorophyll content, biomass and grain yield	Multi environment GWAS integrating physiological trait BLUEs with high density SNP markers (BLINK model)	Marker-assisted selection and physiological trait based breeding for drought and heat resilient wheat cultivars	Devate et al. (2022)
Maize (*Zea mays* L.)	Genomics (GWAS; GBS + DArT SNPs)	Field based phenotyping	Seedling emergence rate, seedling plant height and grain yield (BLUE values)	Multi-environment GWAS using mixed linear model (EMMAX)	Marker-assisted selection for early stage drought resilient maize cultivars	Chen et al. (2023)
Rose (*Rosa hybrida* and *Rosa damascena*)	Transcriptomics, proteomics and metabolomics	Physiological and anatomical phenotyping	Phenylpropanoid and flavonoid biosynthesis, ROS accumulation, leaf anatomy, secondary metabolism regulation and soluble protein content	Multi-omics integration with WGCNA, gene-protein-metabolite network analysis and functional validation (dual luciferase assays)	Identification of regulatory genes (e.g. *CHS1*) and metabolic markers for breeding salt tolerant, high quality ornamental rose cultivars	Ren et al. (2024)
Wheat (*Triticum aestivum*)	Genomics (GWAS using SNP array)	UAV-based multispectral imaging (RGB, Red-edge, NIR) and SPAD validation	Chlorophyll content dynamics across heading, flowering, and grain filling stages	Machine learning assisted phenotype prediction combined with GWAS (MLM: Q+K)	Enables high-throughput field phenotyping, improved locus discovery and marker-assisted selection for drought resilient and photosynthetically efficient wheat genotypes	Cheng et al. (2025)
Rice (*Oryza sativa*)	Transcriptomics (RNA-seq) and Metabolomics (LC-MS/MS)	Morphological and physiological phenotyping	Growth traits, chlorophyll content, gas exchange parameters, chlorophyll fluorescence traits (Fv/Fm), ion homeostasis indicators (Na^+^/K^+^ ratio), oxidative stress markers (H_2_O_2_) and membrane stability indicators (electrolyte leakage)	Comparative multi-omics integrated with pathway and network analysis	Identification of salt tolerance mechanisms and candidate genes from wild rice for introgression and breeding of salt resilient rice cultivars	Deng et al. (2025)
Maize (*Zea mays* L.)	Transcriptomics and Metabolomics	Morphological and physiological phenotyping	Growth traits (Shoot length, root length and biomass) and antioxidant enzymes	Joint DEG-DEM analysis with KEGG/GO pathway co-enrichment across transcriptome and metabolome datasets	Identification of candidate genes, metabolites, and regulatory pathways for breeding salt tolerant maize cultivars adapted to saline soils	Ren et al. (2025)
Potato (*Solanum tuberosum*)	Transcriptomics, Proteomics, Metabolomics and Hormonomics	RGB imaging, chlorophyll fluorescence imaging and thermal IR imaging (PlantScreen platform)	Plant growth traits, photosynthetic performance traits, canopy temperature, water use efficiency and tuber yield components	Machine learning assisted feature selection (random forest + RFE), multivariate correlation and canonical correlation analyses and knowledge based metabolic signaling network integration	Identification of candidate genes, pathways and early phenotypic indicators for breeding potato cultivars with improved resilience to heat, drought, waterlogging and their combinations	Zagorščak et al. (2025)

Genome wide association studies (GWAS) have been crucial in understanding the intricate genetic architecture of crop tolerance to different abiotic stresses. Forty-four significant SNPs linked to yield related traits and Na^+^/K^+^ homeostasis were identified in a GWAS of 220 different indica rice accessions evaluated for field salinity stress during the reproductive stage (Kumar et al., 2015). These included prevalent linkages within the well characterized *Saltol* locus on chromosome 1, as well as novel QTLs on chromosomes 4, 6 and 7, which together accounted for 5-18% of phenotypic variance. These loci represent valuable genomic tools for developing salinity tolerant rice cultivars using marker-assisted technologies Similarly, GWAS of 180 diverse rice accessions evaluated under controlled saline conditions identified 28 significant marker-trait associations, predominantly linked to ionic balance traits. Many such loci were found to be co-localizing with known salt tolerance regions and ion transporter related genes (Warraich et al., 2020). The genetic basis of yield stability under abiotic stresses in wheat has been elucidated through the integration of GWAS with multi-environment phenotyping. Stable QTL hotspots on chromosomes 2A and 2B linked to grain yield, yield components, NDVI and several stress tolerance indices were found in durum wheat through GWAS carried out across yield potential, drought and heat stress environments. These loci identified stress specific and shared genetic controls, offering promising targets for marker-assisted selection targeted at enhancing resistance to heat and drought (Sukumaran et al., 2018).

Linking high-throughput physiological phenotyping with GWAS in wheat for traits such as NDVI, canopy temperature, chlorophyll content and yield across multiple locations identified 57 significant SNPs. These included stable and pleiotropic loci associated with adaptive traits such as biomass maintenance and stay green behavior under combined drought and heat stress, showing the potential of combining physiological phenotyping in the field with genomics (Devate et al., 2022).

Embedding high-throughput phenotyping platforms within GWAS frameworks has further improved the locus discovery in crops. In a multi environment study, 119 diverse winter wheat genotypes were evaluated across two locations under normal irrigation and drought stress conditions. Multispectral UAV imaging was conducted at the heading, flowering and grain filling stages capturing reflectance in red, red edge, near infrared, green and blue bands. Eighteen vegetation indices were screened using a random forest algorithm and the most informative indices were incorporated into a backpropagation neural network model to predict SPAD based chlorophyll content.

The UAV derived predictions showed strong agreement with manual measurements, with correlation coefficients ranging from 0.88 to 0.96 depending on growth stage and environment. These predicted phenotypes together with the measured values were integrated into a mixed linear model (MLM, Q + K) GWAS framework using 36,873 SNP markers. A total of 308 loci explaining 7.58%–19.58% of phenotypic variation were detected across 21 chromosomes, in which UAV predicted phenotypes identified 206 loci in comparison to the 102 loci detected using manually measured values. Eighteen loci were found to overlap between the two datasets. The predicted traits showed smaller average P values and higher proportions of explained variation, indicating improved statistical power for locus discovery. This study illustrates how deep phenotyping combined with machine learning assisted trait extraction can enhance genetic resolution in field scale breeding populations (Cheng et al., 2025). GWAS approaches have also been adapted to investigate other major crops to unravel their genetic mechanisms of abiotic stress tolerance. In upland cotton (*Gossypium hirsutum*), GWAS on 419 diverse genotypes evaluated under seedling stage salt stress identified 23 SNPs and key candidate genes associated with tolerance traits such as relative water content and fresh weight, supported by functional validation using qRT-PCR (Yasir et al., 2019). These loci provide molecular markers for improving cotton productivity on saline soils. The genetic architecture of drought tolerance in maize under field conditions studied through GWAS identified drought responsive SNPs and candidate genes associated with seedling emergence, plant height and grain yield, revealing key pathways involved in transcriptional regulation and metabolism (Chen et al., 2023). These drought responsive loci provide robust genomic resources for marker-assisted selection.

Beyond genomics, abiotic stress tolerant crop phenotypes have resulted from the integration of omics techniques (Jogaiah et al., 2013). Integrated multi-omics approaches combined with physiological profiling have greatly improved the understanding of mechanisms of abiotic stress tolerance in crops. Comparative analysis of transcriptome and metabolome profiles of salt tolerant wild rice line HD96–1 and salt sensitive line IR29 has revealed that coordinated regulation of ion homeostasis, photosynthetic stability, osmotic adjustment and oxidative stress responses mediated by key regulators such as *NHX4* and *ACX4* enhanced salt tolerance in the crop (Deng et al., 2025). Similarly, integrated transcriptomics (RNA-seq) and LC-MS based metabolomics profiling in a salt-tolerant maize inbred line J1285 evaluated under salt stress condition revealed coordinated regulation of ABA and MAPK signaling pathways, redox homeostasis, and phenylpropanoid metabolism, supported by joint DEG (differentially expressed genes) - DEM (differentially expressed metabolites) pathway co-enrichment analysis (Ren et al., 2025). Multi-omics integration combined with detailed physiological characterization has also been applied to horticultural crops such as rose (*Rosa hybrida*). Combined transcriptomic, proteomic, and metabolomic analyses of two rose genotypes under salt stress conditions revealed enhanced phenylpropanoid and flavonoid metabolism as key determinants of salt tolerance, with *CHS1* identified as the central regulatory node through co-expression network analysis and functional validation. Hence, *CHS1* can act as candidate target for breeding salt-resilient roses (Ren et al., 2024).

Integrated multi-omics combined with automated deep phenotyping has recently been applied to dissect the response of potato (Solanum tuberosum) to single and combined abiotic stresses under controlled yet high-throughput conditions. In a comprehensive systems level study, potato plants were exposed to heat (30 °C day/28 °C night), moderate drought (30% field capacity), waterlogging and sequential stress combinations. Morpho physiological traits including plant volume, canopy temperature (ΔT), chlorophyll fluorescence parameters (Fv/Fm_Lss, qL_Lss), compactness and water consumption were quantified daily using the PlantScreen phenotyping platform. Parallel multi-omics profiling was performed on leaf samples collected at early (1 day) and prolonged (7–14 days) stress timepoints. The molecular datasets comprised untargeted proteomics (4,258 proteins identified), targeted transcript profiling of stress and tuberization related genes, metabolomics (22 primary metabolites) and hormonomics (ABA, JA, SA, IAA and derivatives). To enable cross level integration, a machine learning based feature selection approach (random forest with recursive elimination) was used, which reduced phenomics variables to the most informative six traits, while proteomics data were filtered to stress responsive proteins linked to photosynthesis, hormone metabolism, ROS signaling and primary metabolism.

Subsequent statistical modeling, correlation analysis across omics layers and canonical correlation analysis were combined with the construction of a customized knowledge based metabolic and signaling network integrating KEGG derived pathways with experimentally derived data. Overlaying multi-omics measurements onto this network revealed stress specific regulatory modules. Rapid ABA and JA accumulation, ROS signaling and strong repression of photosynthetic efficiency were induced by waterlogging, whereas combined heat and drought stress exhibited a distinct signature characterized by branched chain amino acid accumulation, heat shock protein enrichment and altered tuberization signaling (SP6A modulation). Prolonged stress treatments significantly reduced harvest index and starch accumulation, particularly under waterlogging and combined stresses. Collectively, this framework demonstrates the importance of synchronized deep phenotyping and multi-omics integration in resolving temporal and combinatorial stress signatures, identifying candidate hormonal and metabolic markers, and providing mechanistic targets for climate adapted potato breeding (Zagorščak et al., 2025).

### 5.2 Integrated strategies for biotic stress resilience

Climate change is exacerbating biotic stresses by changing the dynamics of pathogens and pests, which makes crop losses more complex and unpredictable (Thingujam et al., 2025). Integrated multi-omics and deep phenotyping strategies provide a systems level framework for connecting quantitative disease and pest phenotypes with molecular defence responses (**Table 3**). By linking phenotypes with underlying molecular pathways, these methods make it easier to find resilient, network-based resistance mechanisms that can withstand changes in the environment as well as evolution of the pathogen (Varadharajan et al., 2025).

**Table 3:** Applications of integrated omics and phenotyping strategies for improving biotic stress resilience in major crops.

Crop	Omics layer(s)	Phenotyping level	Key pathways/traits resolved	Integration strategy	Application in crop improvement	Reference
Rice (*Oryza sativa* L.)	Transcriptomics (microarray) and Metabolomics (LC–TOF–MS, GC–TOF–MS)	Molecular and biochemical phenotyping	Defense related metabolic pathways (phenylpropanoid, alkaloid, glutathione and amino acid metabolism) and PR gene induction	Systems level integration using multivariate metabolomics (PCA, PLS and HCA) combined with DEG-metabolite pathway co-interpretation	Molecular pathway discovery to support resistance gene deployment and systems guided breeding for bacterial blight resistance (*Xanthomonas oryzae* pv. *oryzae*)	Sana et al. (2010)
Sugar beet (*Beta vulgaris* L.)	Genomics (QTL mapping) and Phenomics	Hyperspectral imaging (460–850 nm; lesion level and temporal)	Spectral signatures of lesions, lesion substructure (center, margin and transition zones), PSSRa, PRI and pixel wise lesion dynamics	Integration of hyperspectral phenomics with QTL defined genotypes using unsupervised spherical k-means clustering and temporal lesion dynamics analysis	High resolution phenotyping to quantify QTL effects and improve selection efficiency for quantitative Cercospora leaf spot disease (*Cercospora beticola*) resistance in sugar beet breeding	Leucker et al. (2016a)
Sugar beet (*Beta vulgaris* L.)	Phenomics (spectral-spatial data)	Hyperspectral imaging (400–900 nm; leaf and lesion level)	Lesion size, center to margin ratio, lesion subarea composition and sporulation density	Pixel wise hyperspectral analysis combined with Spectral Angle Mapper (SAM) based lesion subarea classification and validated using microscopic imaging and sporulation quantification	High-throughput, non-destructive phenotyping to improve screening and selection for cercospora leaf spot resistance (*Cercospora beticola*) in sugar beet breeding	Leucker et al. (2016b)
Wheat (*Triticum aestivum* L.)	Phenomics	UAV-mounted hyperspectral imaging (400–900 nm)	Disease severity classes, spectral reflectance signatures and vegetation indices (GNDVI, PRI, RVSI and Chl green)	Machine learning based integration of UAV hyperspectral phenomics using feature selection and multi class classification (Random Forest, QDA, SVM, DTC), validated against disease severity scores	High-throughput, non-destructive phenotyping for early stem rust (*Puccinia graminis* f. sp. *tritici*) detection and resistance screening in wheat breeding	Abdulridha et al. (2023)
Wheat (*Triticum aestivum* L.)	Genomics (GBS-based GWAS)	Hyperspectral imaging of wheat kernels (397–1004 nm; Specim IQ handheld system)	Mean kernel reflectance values (204 wavebands), PC1 of full hyperspectral phenome, PC1 of sliding-window spectral bins, GC/MS derived DON	PCA based dimensionality reduction (full spectrum and sliding windows) with GWAS using the BLINK model	High-throughput, phenomics enabled discovery of Fusarium head blight (*Fusarium graminearum*) resistance loci for breeding wheat varieties with reduced DON mycotoxin accumulation	Concepcion et al. (2024)
Wheat (*Triticum aestivum* L.)	Phenomics	Leaf and canopy level hyperspectral reflectance spectroscopy (450–2400 nm)	Disease severity classes and spectral signatures linked to pigment loss and water content	Machine learning integration (Random Forest) with dimensionality reduction (PCA, backward feature elimination)	High-throughput disease phenotyping to aid selection of stripe rust (*Puccinia striiformis* f. sp. *tritici*) resilient germplasm and precise disease monitoring	Cross et al. (2024)
Soybean (*Glycine max* L.)	Transcriptomics (RNA-seq) and Metabolomics (UHPLC-MS/MS)	Molecular and biochemical phenotyping	Isoflavonoid and phenylpropanoid biosynthesis, branched chain amino acid metabolism, MAPK signaling, phytohormone signaling (JA, BR) and NLR mediated immune responses	KEGG-based DEG-DAM pathway integration, O2PLS multivariate analysis, gene-metabolite correlation networks and temporal transcriptome profiling	Systems guided discovery of molecular markers and pathways to enhance resistance to phytophthora root rot (*Phytophthora sojae*) in soybean	Sun et al. (2024)
Tea (*Camellia sinensis*)	Transcriptomics (RNA-seq) and Metabolomics (UPLC-ESI-MS/MS, GC-MS)	Hyperspectral imaging (397–800 nm; leaf and canopy layer)	Spectral disease signatures, canopy layer infection severity and diagnostic wavelengths (552, 673 and 800 nm)	Integration of hyperspectral phenotyping with transcriptome-metabolome co-analysis, including DEG-DAM pathway enrichment and WGCNA based gene-metabolite co-expression network construction	Development of non-destructive disease detection and canopy stratified phenotyping coupled with identification of candidate genes and metabolites for breeding sooty mold (*Cladosporium* spp.) resistant tea cultivars	Wang et al. (2024)

Evidence from integrated transcriptomic and metabolomic studies have provided important insights into the molecular networks that shape biotic stress resistance in crops. Parallel application of LC–TOF/GC–TOF metabolomics and microarray-based transcriptomics to dissect *Xa21* mediated resistance against bacterial leaf blight in rice uncovered coordinated activation of phenylpropanoid and alkaloid biosynthesis, glutathione metabolism and pathogenesis-related genes, with resistant genotypes exhibiting clear metabolic reprogramming relative to susceptible ones. These broader pathway-level signatures have direct relevance in breeding for durable bacterial leaf blight resistance in rice (Sana et al., 2010).

Approaches combining genetic mapping with deep phenotyping technologies capable of resolving quantitative disease traits with high precision enable high resolution dissection of quantitative disease resistance. For instance, in sugar beet, hyperspectral imaging-based temporal phenotyping of cercospora leaf spot, combined with QTL-mapping, revealed that resistance loci influence not only overall disease severity but also lesion expansion dynamics, spectral characteristics, and lesion substructure. These QTL effects were largely undetectable using conventional visual scoring, demonstrating the sensitivity of hyperspectral phenomics in identifying minor but biologically significant resistance characteristics (Leucker et al., 2016a). At an even finer scale, lesion-level hyperspectral imaging of sugar beet leaves infected with *Cercospora beticola* enabled the discrimination of distinct lesion phenotypes and sub-lesion zones. Resistant genotypes were characterized by smaller lesion centers, reduced sporulation, and unique spectral signatures compared with susceptible lines (Leucker et al., 2016b). The study shows the capacity of high-resolution hyperspectral phenotyping to capture quantitative resistance components and thereby improve the screening efficiency and accelerated development of resistant cultivars.

The utility of deep phenotyping has been further amplified through the integration of machine learning, particularly for large-scale field assessments. In wheat, UAV based hyperspectral imaging combined with supervised classification algorithms enabled accurate discrimination of stem rust severity across extensive field trials, including the detection of early infection stages that were visually indistinguishable (Abdulridha et al., 2023). By exploiting informative spectral bands and vegetation indices such as GNDVI, PRI, and RVSI, this approach achieved classification accuracies of up to 85%, demonstrating its potential for high-throughput disease resistance screening under field conditions. Complementary work using visible to shortwave infrared hyperspectral reflectance and machine learning further enabled precise quantification of wheat stripe rust progression at both leaf and canopy scales, with accuracies approaching 96%, while identifying spectral regions associated with pigment degradation and plant water status during early disease development (Cross et al., 2024).

Deep phenotyping of fusarium infected kernels in wheat using hyperspectral imaging coupled with GWAS identified a major effect QTL on chromosome 2D that influenced the hyperspectral phenome and was associated with a significant reduction in deoxynivalenol (DON) accumulation (Concepcion et al., 2024). In this study, hyperspectral principal components explained up to 26% of the phenotypic variance, demonstrating that imaging derived traits can serve as informative correlated phenotypes for uncovering resistance loci that may be difficult to detect using conventional disease scores. Multi-omics technologies such as integrated transcriptomic and metabolomic analyses continue to provide insights into disease resistance pathways in crops. For example in soybean, time resolved RNA-seq and LC–MS based metabolomics analyses of transgenic lines overexpressing an NLR gene post *Phytophthora sojae* infection revealed coordinated activation of isoflavonoid and phenylpropanoid biosynthesis, branched chain amino acid metabolism, MAPK signaling, and phytohormone pathways (Sun et al., 2024). Resistant lines in the study displayed pronounced metabolic reprogramming, with isoflavonoids emerging as key metabolites associated with resistance and promising molecular markers for breeding cultivars with enhanced resistance to Phytophthora root rot.

The highest resolution of biotic stress responses can be achieved through the integration of deep phenotyping with multi-omics within a unified systems biology framework. Hyperspectral imaging combined with transcriptomic and metabolomic profiling in tea (*Camellia sinensis*) enabled spatially resolved discrimination of sooty mold severity across canopy layers. Identification of D-mannitol and mannitol dehydrogenase as key hub components, highlights coordinated regulation of secondary metabolite biosynthesis, hormone signaling, and plant-pathogen interaction pathways in the study. These findings can collectively benefit disease resistance breeding strategies and provide a framework for precision phenotyping (Wang et al., 2024).

The advent of integrated multi-omics and deep phenotyping approaches is redefining the landscape of how plants respond to both abiotic and biotic challenges anticipated in the context of a changing climate. The power of these approaches lies in their ability to integrate high-resolution molecular data with dynamic or quantitative information in terms of phenotype in a manner not possible through single layer analyses. The recent research studies have made it abundantly clear the relevance of integrated approaches in the realm of multi-omics studies, advanced approaches in phenotyping, as well as data driven analytics in supporting more predictive and resilient breeding pipelines.

## 6 Limitation and challenges

The application of modern and advanced technologies like multi-omics and deep phenotyping poses serious constraints and challenges that need to be overcome to use them in crop improvement. Among the most significant challenges, one can identify the possibility to handle the enormous number and depth of information that multi-omics and deep phenotyping platforms generate, which involves the huge numbers of genomic, transcriptomic, proteomic, metabolomic, ionomic, and complex phenotypic data (Xu, 2016). It is computationally challenging and requires thorough biological understanding to combine these heterogeneous datasets into a unified and understandable framework and that preliminary preprocessing challenges, including normalization, transformation, and scaling, are additional complicators of integration work (Mahmood et al., 2022). Moreover, the sufficient control, management, search, mapping, and reporting of inter-relationships between genes, proteins, and metabolites is a significant bottleneck (Raza et al., 2024). Although it has already been in the arena of genomics, the main objective of addressing the existing genotype-to-phenotype gap (especially in diverse and complex stress conditions) is yet to be reached even in advanced breeding programs (Razzaq et al., 2021). Also contributing to this gap are environmental variability and phenotypic plasticity. In addition, high-throughput phenotyping platforms are expensive to maintain and require large amounts of financial investment and are often highly tailored and expensive, so that not all people can access them, particularly in resource-limited environments, and they contribute to an increasing digital divide (Poorter et al., 2023; Parra-López et al., 2024). The complexity and cost of the systems are further exacerbated by the fact that by simulating the world of climate change, a variety of growth environments will be employed. Reproducibility and cross-study comparability are also hampered by the missing standardized experimental designs, calibration procedures, and generally adopted protocols. Moreover, it is still difficult to interpret multidimensional multi-omics data and convert it into biologically significant and practically usable results, which require the use of complex bioinformatics software, powerful analytical models, and expert skills to create prediction models and facilitate their translation to the field level (Amin et al., 2025a). In the absence of these structures, the huge amount of information produced may tend to overwhelm the researchers and restrict the actionable information (Xu, 2016), whereas the lack of field phenotyping infrastructure in most, especially developing countries, reduces the usefulness of the research findings even more (Varshney et al., 2012). Therefore, regardless of the enormous opportunities, data integration issue, the genotype-phenotype gap, and high costs of technology, and the necessity of standard approaches and robust analytic pipelines, multi-omics and deep phenotyping represent unprecedented opportunities, but the current extent of its use in crop improvement depends on collaborative research and capacity building due to technological advantages and technological disadvantages in a climate-resilient society.

## 7 Conclusion and future directions

The convergence of multi-omics technologies with deep phenotyping, using association mapping and machine learning has begun to transform the understanding of climate resilience in crops by linking molecular variation to dynamic physiological performance across multiple environments. Integrated genomics, transcriptomics, proteomics, metabolomics, ionomics, and high-resolution phenomics have revealed stress-responsive networks, regulatory hubs, and diagnostic biomarkers that were inaccessible to single-layer analyses, thereby enabling more informed trait dissection and selection in both field and controlled conditions. Together with association mapping and genomic prediction, these approaches are already delivering stress-resilient genotypes in major crops such as wheat, rice, maize, potato, soybean, and cotton, thereby demonstrating the practical utility.

Despite the progress, substantial bottlenecks continue to limit the routine deployment of integrated multi-omics and deep phenotyping pipelines in breeding. The extreme heterogeneity, dimensionality, and scale of omics and imaging datasets impose stringent demands on data management, curation, and harmonization, while batch effects, incomplete metadata, and divergent experimental designs hinder cross-study comparability and meta-analytic synthesis. High-throughput phenotyping platforms and advanced imaging systems remain expensive and, thereby widening the technological and capacity gap in multiple research sectors. Furthermore, translating complex, model-derived features into biologically meaningful, breeder-friendly decision rules remains challenging, reinforcing the genotype–phenotype gap under variable field conditions.

Machine learning and deep learning frameworks provide powerful tools for extracting structure from multi-omics and phenomics data, improving phenotype prediction, and capturing nonlinear genotype–environment–management interactions. Supervised, unsupervised, kernel-based, hybrid, AutoML, and ensemble models have already improved the prediction of yield, stress indices, and disease severity using integrated molecular and imaging traits, often outperforming conventional statistical approaches. However, issues related to limited sample sizes, label noise, model overfitting, algorithmic bias, and limited interpretability constrain their wider adoption in breeding decision pipelines. Addressing these limitations will require transparent model design, rigorous validation across environments and genetic backgrounds, and closer embedding of biological priors and network knowledge into model architectures to avoid purely black-box prediction.

The next generation of climate-resilient crop improvement will depend on building interoperable, compliant data that integrates multi-omics, 3D and 4D phenomics, environmental, and management information across scales and years. Standardized experimental designs, ontologies, and analysis workflows will be essential for enabling robust cross-site integration, benchmarking of models, and reuse of sample data beyond individual research. Integration of cloud-native infrastructures, edge computing, and scalable analytical pipelines to phenotyping platforms, can help in reducing the entry barriers for breeders around the globe. Future research should prioritize interpretable, biologically grounded machine learning models that couple graph-based representations, causal inference, and mechanistic simulation with multi-omics and phenotyping data. Such models can reveal regulatory modules, key metabolites, and structural traits underpinning resilience and provide actionable targets for genome editing, marker-assisted selection, and genomic selection. Similar efforts to expand research in these sectors in underutilized and orphan crops, integrating plant–microbiome omics, and incorporating socio-economic dimensions will further widen the benefits of these integrative technologies. Ultimately, the integration of multi-omics, deep phenotyping, and AI-driven analytics offers a robust framework to accelerate the discovery of adaptive traits, shorten breeding cycles, and deliver climate-resilient, high-yielding, and nutritious cultivars at scale. Realizing this potential and its quick implementation in multiple crops will depend on sustained infrastructural investments, ensuring open data access, interdisciplinary training, and international collaborations that bridge gaps in technological advancements, existing breeding pipelines and farmers’ needs.
